# ATG16L1 restrains macrophage NLRP3 activation and alveolar epithelial cell injury during septic lung injury

**DOI:** 10.1002/ctm2.70289

**Published:** 2025-04-11

**Authors:** Yan Bai, Xinyu Zhan, Qing Zhu, Xingyue Ji, Yingying Lu, Yiyun Gao, Fei Li, Zhu Guan, Haoming Zhou, Zhuqing Rao

**Affiliations:** ^1^ Department of Anesthesiology The First Affiliated Hospital of Nanjing Medical University Nanjing China; ^2^ Hepatobiliary Center The First Affiliated Hospital of Nanjing Medical University, Key Laboratory of Liver Transplantation, Chinese Academy of Medical Sciences, NHC Key Laboratory of Living Donor Liver Transplantation, Nanjing Medical University Nanjing China

**Keywords:** acute lung injury, ATG16L, macrophages, NLRP3 inflammasome, STING

## Abstract

**Background:**

The lung is the organ most commonly affected by sepsis. Additionally, acute lung injury (ALI) resulting from sepsis is a major cause of death in intensive care units. Macrophages are essential for maintaining normal lung physiological functions and are implicated in various pulmonary diseases. An essential autophagy protein, autophagy‐related protein 16‐like 1 (ATG16L1), is crucial for the inflammatory activation of macrophages.

**Methods:**

ATG16L1 expression was measured in lung from mice with sepsis. ALI was induced in myeloid *ATG16L1‐*, *NLRP3‐* and *STING*‐deficient mice by intraperitoneal injection of lipopolysaccharide (LPS, 10 mg/kg). Using immunofluorescence and flow cytometry to assess the inflammatory status of LPS‐treated bone marrow‐derived macrophages (BMDMs). A co‐culture system of BMDMs and MLE‐12 cells was established in vitro.

**Results:**

Myeloid *ATG16L1*‐deficient mice exhibited exacerbated septic lung injury and a more intense inflammatory response following LPS treatment. Mechanistically, *ATG16L1*‐deficient macrophages exhibited impaired LC3B lipidation, damaged mitochondria and reactive oxygen species (ROS) accumulation. These abnormalities led to the activation of NOD‐like receptor family pyrin domain‐containing protein 3 (NLRP3), subsequently enhancing proinflammatory response. Overactivated *ATG16L1*‐deficient macrophages aggravated the damage to alveolar epithelial cells and enhanced the release of double‐stranded DNA (dsDNA), thereby promoting STING activation and subsequent NLRP3 activation in macrophages, leading to positive feedback activation of macrophage NLRP3 signalling. Scavenging mitochondrial ROS or inhibiting STING activation effectively suppresses NLRP3 activation in macrophages and alleviates ALI. Furthermore, overexpression of myeloid *ATG16L1* limits NLRP3 activation and reduces the severity of ALI.

**Conclusions:**

Our findings reveal a new role for ATG16L1 in regulating macrophage NLRP3 feedback activation during sepsis, suggesting it as a potential therapeutic target for treating sepsis‐induced ALI.

**Key points:**

- Myeloid‐specific ATG16L1 deficiency exacerbates sepsis‐induced lung injury.
- *ATG16L1*‐deficient macrophages exhibit impaired LC3B lipidation and ROS accumulation, leading to NLRP3 inflammasome activation.
- Uncontrolled inflammatory responses in *ATG16L1*‐deficient macrophages aggravate alveolar epithelial cell damage.
- Alveolar epithelial cells release dsDNA, activating the cGAS‐STING‐NLRP3 signaling pathway, which subsequently triggers a positive feedback activation of NLRP3.
- Overexpression of *ATG16L1* helps mitigate lung tissue inflammation, offering a novel therapeutic direction for sepsis‐induced lung injury.

## Graphical abstract

1. *ATG16L1‐*deficient macrophages exhibited impaired LC3B lipidation and accumulation of ROS, resulting in the activation of the NLRP3 inflammasome.
2. Overactivated *ATG16L1*‐deficient macrophages aggravated the damage of alveolar epithelial cells, and increased the release of dsDNA.
3. Released dsDNA activated the STING‒NLRP3 signalling pathway, leading to positive feedback activation of macrophage NLRP3 signalling in macrophages.

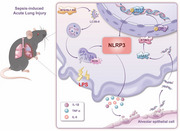

## 1 INTRODUCTION

Sepsis is a severe inflammatory response caused by infection, which can lead to multiple organ failure and death.^1^ In 2017, sepsis was estimated to cause 11 million deaths, accounting for nearly 20% of all global fatalities.^2^ Bacteria have been identified as the primary pathogens causing infection‐induced sepsis.^3^ Lung is the most vulnerable organs during sepsis, with over 50% of patients with sepsis developing ALI.^4^ The primary characteristic of sepsis is immune dysregulation triggered by infection, which often manifests as excessive inflammation.^5^ During early stages of infection, various stimuli triggers the activation of pattern recognition receptors, prompting macrophages, dendritic cells and neutrophils to release inflammatory cytokines.^6^ In later stages, some patients exhibit persistent inflammation, immunosuppression and catabolic syndrome, which are characterised by prolonged hyperinflammation, immunosuppression and bone marrow dysfunction.^7^ Acute respiratory distress syndrome is a severe and progressive complication of sepsis, and has a high mortality rate.^8^

NLRP3 inflammasome is a well‐studied supramolecular complex whose activation coordinates the cleavage of pro‐caspase1 and secretes cytokines, subsequently triggering a robust immune response.^9^ Notably, the macrophage NLRP3 activation significantly contributes to mice ALI. The inhibition of NLRP3 can markedly alleviate lung injury.^10^, ^11^

Autophagy is a process of cellular self‐digestion through which cells degrade and recycle damaged organelles and proteins, thereby maintaining cellular homeostasis,^12^ which facilitates the clearance of microorganisms and controls the inflammatory response by negatively regulating macrophage inflammasome activation.^13^, ^14^ ATG16L1 is essential for autophagy, aiding in autophagosome formation and maturation. It binds to ATG5 and recruits the ATG12‒ATG5 complex to the site of autophagosome formation.^15^ The ATG12‒ATG5‒ATG16L1 complex possesses E3 ligase activity, facilitating the recruitment of LC3 to the phagophore and activating the E2 ligase ATG3, which in turn mediates the conjugation of LC3 with phosphatidylethanolamine (PE).^16^, ^17^ Studies have indicated that the absence of ATG16L1 impairs the recruitment of the Atg12‒Atg5, leading to a failure in LC3 conjugation to PE.^18^ IL‐22 stimulation of *ATG16L1*‐deficient intestinal epithelial cells induces endoplasmic reticulum stress and necroptosis, subsequently activating STING signalling and inducing ileitis in vivo.^19^ Furthermore, *ATG16L1*‐deficient macrophages exhibited defective autophagy and promoted the secretion of inflammatory cytokines upon LPS stimulation.^18^ However, the specific role of ATG16L1 in macrophages and its mechanisms of action in sepsis‐induced lung injury remains poorly understood.

Herein, the critical role of ATG16L1 in controlling lung tissue inflammation has been confirmed. Mechanistically, our study revealed that *ATG16L1*‐deficient macrophages are involved in the regulation of molecules such as NLRP3 and STING, resulting in an uncontrolled inflammatory response and subsequent macrophage activation in the lung tissues.

## 2 MATERIALS AND METHODS

### 2.1 Mice studies

Wild‐type (WT), FloxP‐*atg16l1* (*ATG16L1^fl/fl^*), myeloid‐specific *atg16l1*‐knockout (*ATG16L1^mKO^*), myeloid‐specific *atg16l1*‐overexpressing‐knockin (*ATG16L1^mKI^*), FloxP‐*nlrp3* (*NLRP3^fl/fl^*) and myeloid‐specific *nlrp3*‐knockout (*NLRP3^mKO^*) C57BL/6 male mice were engineered by GemPharmatech Co. FloxP‐*Tmem173* (*STING^fl/fl^*) and myeloide‐specific *Tmem173*‐knockout (*STING^mKO^*) male mice on a C57BL/6 background were engineered by Shanghai Model Organisms Center. The *ATG16L1^fl/fl^*, *NLRP3^fl/fl^* and *STING^fl/fl^* mice served as WT controls. All mice were housed in a standard, specific pathogen‐free environment. Animal procedures complied with the legal and ethical guidelines set forth in the protocol approved by the Institutional Animal Care and Use Committee of Nanjing Medical University (IACUC‐2402019).

Mice were intraperitoneally injected with LPS (10 mg/kg; Sigma‒Aldrich) to establish the model of sepsis‐induced ALI. The sham group was injected intraperitoneally with an equal amount of phosphate buffer saline (PBS). A dose of LPS (20 mg/kg) was administered through intraperitoneal injection for the survival studies.

The selective NLRP3 inhibitor MCC950 (10 mg/kg; MedChemExpress) was administered intraperitoneally 4 h before the LPS injection to inhibit the NLRP3 inflammasome. To reduce mitochondrial reactive oxygen species (ROS) production, Mitotempo (20 mg/kg, Sigma), a mitochondria‐specific antioxidant, was given via intraperitoneal injection. To inhibit anti‐8‐hydroxydeoxyguanosine (8‐OHdG), mice received an intraperitoneal injection of anti‐8‐OHdG antibody (10 mg/kg; GeneTex) 2 h before the LPS injection, while the control group was administered immunoglobulin G (5 mg/kg; R&D Systems).

### 2.2 BMDMs culture and treatments

As previously mentioned, BMDMs were isolated from the mice and cultured in Dulbecco's Modified Eagle Medium(DMEM) medium containing colony‐stimulating factor (CSF).^20^ LPS (100 ng/mL) stimulated BMDMs for 12 h. In in vitro experiments, BMDMs were pretreated with Mitotempo (1 mM; MedChemExpress), MCC950 (10 µM; MedChemExpress), 3‐MA (5 Mm; MedChemExpress) and C‐176 (20 µM; MedChemExpress) 2 h before LPS stimulation.

### 2.3 NLRP3 siRNA and STING‐siRNA in vivo and in vitro transfection

STING‐siRNA (Santa Cruz Biotechnology) was dissolved in an in vivo transfection reagent and intratracheally administered to mice 48 h prior to LPS treatment. The control group was administered a negative control siRNA using the same vector.

For in vitro transfection of BMDMs, targeting siRNAs for NLRP3 and STING, along with a negative control siRNA (Santa Cruz Biotechnology), were dissolved in OPTI‐MEM medium. Prior to transfection, BMDMs were pre‐incubated in six‐well plates with antibiotic‐free medium for 24 h. Transfection was carried out using Lipofectamine 2000 (Invitrogen) and serum‐free medium upon reaching 60%–80% confluency. After 6 h, fresh medium with 10% fetal bovine serum (FBS) replaced the transfection medium. The BMDMs were then incubated at 37°C in a humidified atmosphere with 5% CO_2_. BMDMs and supernatants were then collected for subsequent Western blot and quantitative real‐time qPCR (RT‐qPCR) analyses.

### 2.4 Histopathology

Lung tissues sections (4 µm thick) were stained with haematoxylin and eosin (H&E). Observe the samples using an optical microscope (Carl Zeiss). Lung tissue damage is assessed by evaluating the degree of haemorrhage, neutrophil infiltration, hyaline membrane formation, persistent debris in the airspaces and septal thickening.^21^

### 2.5 BMDMs and lung tissues immunofluorescence staining

BMDMs were fixed using 4% paraformaldehyde for 15 min. The membranes were treated with. 3% Triton X‐100 for 10 min to permeabilise, followed by a 60‐min blocking step using goat serum. The lung tissue sections were deparaffinised and underwent antigen retrieval, followed by blocking with goat serum for 60 min. Stained BMDMs and lung tissues were observed and imaged on a laser confocal microscope with 10×, 20× and 40× objective lenses (Zeiss LSM 780, Carl Zeiss). Antibodies used are provided in Table S1.

### 2.6 Western blot

BMDMs and lung tissue samples were utilised to extract cellular proteins using RIPA buffer containing protease and phosphatase inhibitors. The supernatant from BMDMs was collected, and proteins were precipitated with methanol and chloroform to isolate the supernatant proteins.^22^ The extracted proteins were separated by SDS/PAGE gels (8%, 10% or 15% [vol/vol]). Antibodies used are provided in Table S2. The ImageJ software was used to perform quantitative and semi‐quantitative analyses of the Western blot results.

### 2.7 Assessment of mitochondrial membrane potential （MMP) and ROS levels

In healthy mitochondria, JC‐10 aggregates in the mitochondrial matrix to form polymers, emitting intense red fluorescence (Ex = 540 nm and Em = 590 nm). In damaged mitochondria, a reduction or loss of membrane potential causes JC‐10 to exist as a monomer in the plasma of stem cells, producing green fluorescence (Ex = 490 nm and Em = 525 nm). MMP was assessed using the JC‐10 kit (Maokang). The measurement of ROS levels in BMDMs and lung tissues was conducted using 2,7‐dichlorodihydrofluorescein diacetate (Yeasen). The measurement was conducted via flow cytometry and immunofluorescence staining.

### 2.8 Measurement of malondialdehyde (MDA), superoxide dismutase (SOD) and the glutathione/glutathione oxidized (GSH/GSSG) levels

Lung tissues were washed with PBS, and the tissue was minced and homogenised by sonication. The tissue was further homogenised and centrifuged at 10 000 × *g* for 10 min to obtain the supernatant. The concentrations of MDA, GSH, GSSG and SOD were determined according to the manufacturer's instructions (Nanjing Jiancheng).

### 2.9 Real‐time qPCR

TRIZOL reagent (Invitrogen) was used to isolate total RNA from lung tissues and BMDMs. The RNA was subsequently converted into cDNA with the assistance of the Takara reverse transcriptase kit (Takara). RT‐qPCR analysis was conducted using the SYBR RT‐PCR kit (Takara). Quantitative PCR was repeated four times. The PCR primer sequences are provided in Table S3. Expression levels of individual genes were normalised to β‐actin as an internal control.

### 2.10 Quantification of 8‐OHdG levels

8‐OHdG in mice serum and co‐culture systems MLE‐12 supernatant were determined using an 8‐hydroxydeoxyguanosine Assay Kit (Nanjing Jiancheng).

### 2.11 Transmission electron microscopy

BMDMs treated with LPS were fixed using electron microscopy fixative (G1102‐100ML, Servicebio). Processed macrophages were analysed using transmission electron microscopy. Imaging was performed using an electron microscope (HITACHI). The images were captured at magnifications of 2.5K× and 7K×.

### 2.12 Establish a co‐culture system of BMDMs and MLE‐12 cells

Mouse alveolar cells (MLE‐12, ATCC) are cultured in the basolateral chamber of a six‐well transwell system, and BMDMs are placed in the apical chamber. After co‐culturing in the system for 24 h, the supernatant from the MLE‐12 cells is collected, and the cells are separated for analysis.

### 2.13 Lactate dehydrogenase activity assay

To assess MLE‐12 cell death, the level of lactate dehydrogenase (LDH) in the supernatant of co‐cultured MLE‐12 cells was measured using a kit in accordance with the manufacturer's protocols (Nanjing Jiancheng).

### 2.14 Statistical analysis

Statistical analysis was conducted using the GraphPad Prism software (version 9.5.1, GraphPad Software Inc.). The Shapiro‒Wilk test was used to assess the normality of the data. Continuous variables with a normal distribution were analysed using either the Student's unpaired *t*‐test or ANOVA with post hoc Tukey's test. For continuous data that are not normally distributed, the Kruskal‒Wallis rank‐sum test is recommended for group comparisons. A ^*^ *p* ≤. 05 (two‐tailed) was regarded as statistically significant.

## 3 RESULTS

### 3.1 Myeloid‐specific *ATG16L1* deficiency exacerbates sepsis‐induced lung injury

**FIGURE 1:**
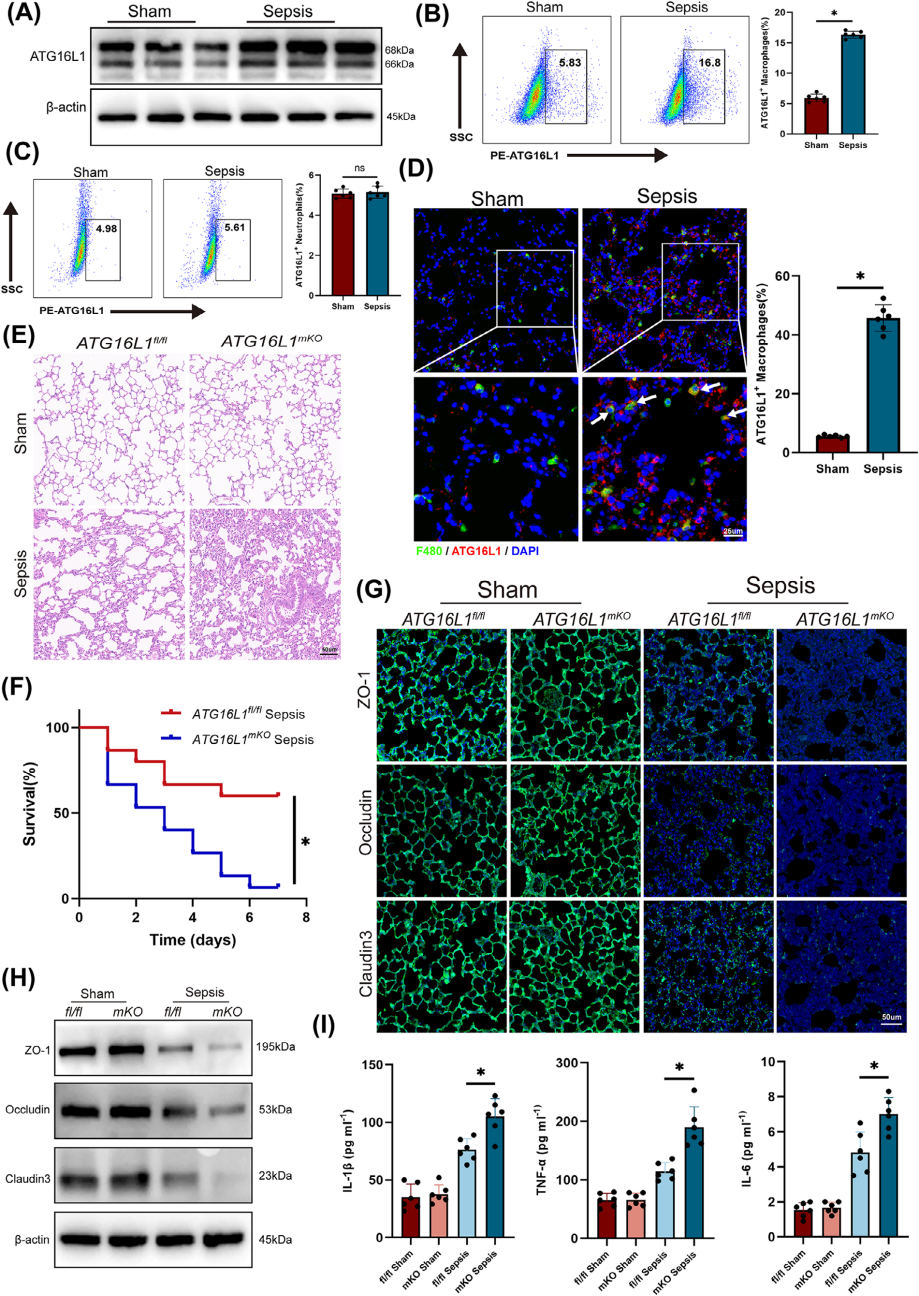
Myeloid‐specific ATG16L1 deficiency exacerbates sepsis‐induced lung injury. ATG16L1*^fl/fl^* and ATG16L1*^mKO^* mice were intraperitoneally injected with lipopolysaccharide (LPS; 10 mg/kg) or PBS. Lung samples and BALF were collected 24 h after the treatment. (A) Protein levels of ATG16L1 in lung tissues of Sham and sepsis groups (*n* = 6/group). (B) Flow cytometry analysis the expression levels of ATG16L1 in alveolar macrophages isolated from BALF. (C) Flow cytometry analysis the expression levels of ATG16L1 in neutrophils isolated from BALF (*n* = 6/group). (D) Representative immunofluorescence images of F4/80 (green) and ATG16L1 (red) in lung tissues. Blue indicates nuclei stained with 4',6‐diamidino‐2‐phenylindole(DAPI). White arrows indicate macrophages with elevated ATG16L1 levels (*n* = 6/group; scale bar: 25 µm). (E) Haematoxylin and eosin (H&E) staining of lung tissue sections (*n* = 6/group; scale bar: 50 µm). (F) Survival analysis of *ATG16L1^fl/fl^* and *ATG16L1^mKO^* mice after LPS (20 mg/kg) treatment (*n* = 15/group). (G) Representative images of immunofluorescence staining of ZO‐1 (green), Occludin (green) and Claudin 3 (green) in lung tissues. Blue indicates nuclei stained with DAPI (*n* = 6/group; scale bar: 50 µm). (H) Western blot analysis of ZO‐1, Occludin, Claudin 3 and β‐actin in lung tissues (*n* = 6/group). (I) Concentration of inflammatory cytokines IL‐1β, TNF‐α and IL‐6 in murine BALF (*n* = 6/group). Data are presented as the mean ± SEM. ^*^ *p* < .05.

Initially, ATG16L1 levels in the lungs of normal and septic mice were examined. Following LPS treatment, the protein levels of ATG16L1 in lungs increased (Figure 1A). Then, the alveolar macrophages and neutrophils were isolated from the BALF following methods previously established by our research group.^23^ Flow cytometry revealed an increase in ATG16L1 levels in alveolar macrophages, while no significant changes were observed in neutrophils (Figure 1B,C). Furthermore, immunofluorescence co‐localisation analysis confirmed that ATG16L1 was upregulated in lung macrophages during sepsis (Figure 1D). We constructed mice with myeloid *ATG16L1* deficiency (Figure S1A). Following LPS treatment, lung tissue H&E staining indicated increased infiltration of inflammatory cells and aggravated damage to the lungs of *ATG16L1^mKO^* mice (Figures 1E and S1D). Compared with the *ATG16L1^fl/fl^* group, the *ATG16L1^mKO^* group had a lower 7‐day survival rate (Figure 1F). Additionally, a significant decrease ZO‐1, Occludin and Claudin 3 levels was observed in the lung tissues of *ATG16L1*‐deficient mice, indicating more severe disruption of the lung tissue barrier structure (Figure 1G,H). The concentrations and mRNA of IL‐1β, TNF‐α and IL‐6 significantly increased in *ATG16L1^mKO^* mice (Figures  and S1E). These results suggest that *ATG16L1*‐deficient mice exhibit a stronger inflammatory response and more severe lung tissue damage following LPS challenge.

### 3.2 *ATG16L1* deficiency promotes macrophage NLRP3 inflammasome activation to aggravate ALI

**FIGURE 2:**
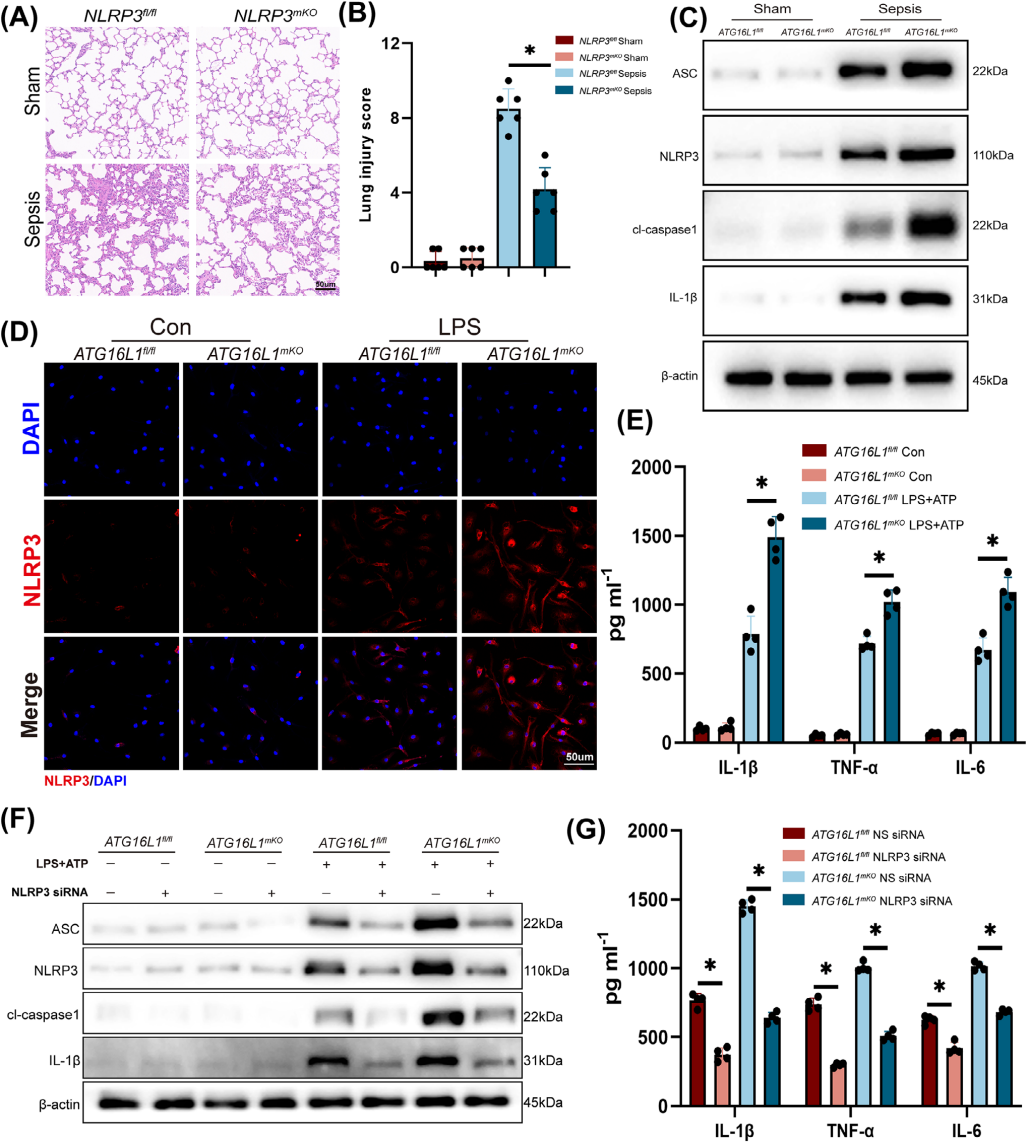
*ATG16L1* deficiency promotes macrophage NLRP3 inflammasome activation to aggravate acute lung injury (ALI). (A and B) *NLRP3^fl/fl^* and *NLRP3^mKO^* mice were intraperitoneally injected with lipopolysaccharide (LPS; 10 mg/kg) or PBS. Lung samples and BALF were collected 24 h after the treatment. Haematoxylin and eosin (H&E) staining of lung tissue sections and lung tissues injury scores (*n* = 6/group; scale bar: 50 µm). (C) Western blot analysis of apoptosis‐associated speck‐like protein containing a CARD (ASC), NLRP3, cl‐caspase1, IL‐1β and β‐actin in lung tissues from LPS‐treated *ATG16L1^fl/fl^* and *ATG16L1^mKO^* mice (*n* = 6/group). (D) Representative images of immunofluorescence staining of NLRP3 (red) immunofluorescence staining. Blue indicates nuclei stained with DAPI (*n* = 4/group; scale bar: 50 µm). (E) Levels of inflammatory cytokines IL‐1β, TNF‐α and IL‐6 in the supernatants of bone marrow‐derived macrophages (BMDMs) (*n* = 4/group). (F) BMDMs were transfected with NLRP3 siRNA or non‐specific siRNA (control) and then stimulated with 100 ng/mL of LPS. Protein levels of ASC, NLRP3, cl‐caspase1, IL‐1β and β‐actin were measured using Western blotting (*n* = 4/group). (G) Levels of inflammatory cytokines IL‐1β, TNF‐α and IL‐6 in the supernatants of BMDMs with NLRP3 siRNA treatment (*n* = 4/group). Data are presented as the mean ± SEM. ^*^ *p* < .05.

Activation of the macrophages NLRP3 results in the release of inflammatory cytokines, which can cause damage to epithelial and endothelial cells.^21^ To further explore the role of NLRP3 in ALI, myeloid‐specific *NLRP3*‐knockout mice were generated (Figure S1B). We confirmed that deletion of myeloid *NLRP3* alleviated inflammation in lung tissues and reduced inflammatory cytokine levels in BALF (Figures 2A,B and S2A). To examine the impact of ATG16L1 on NLRP3 activation, a sepsis model was established, NLRP3 and cleaved‐caspase1 significantly elevated in *ATG16L1*‐deficient mice (Figure 2C). BMDMs stimulated with LPS and ATP markedly enhanced NLRP3 activation (Figures 2D and S2B). The cytokines IL‐1β, TNF‐α and IL‐6 and their mRNA expression in BMDMs supernatants were significantly elevated (Figures 2E and S2C). To investigate the interaction between ATG16L1 and the NLRP3 inflammasome, NLRP3 siRNA was utilised to knock down *NLRP3* in BMDMs. NLRP3 siRNA effectively reduced NLRP3 expression in BMDMs and decreased cl‐caspase1 activation (Figure 2F). Furthermore, the secretion of inflammatory factors was significantly reduced in the supernatant of BMDMs treated with NLRP3 siRNA (Figure 2G). MCC950, an NLRP3 inhibitor, effectively suppressed NLRP3 activation in *ATG16L1*‐knockout BMDMs (Figure S2D). The cytokines IL‐1β, TNF‐α and IL‐6 in BMDMs supernatants were significantly elevated (Figure S2E). MCC950 significantly alleviated lung tissues inflammation and reduced lung injury (Figure S2F,G). These results suggest that *ATG16L1* deficiency amplifies the inflammatory response through the activation of NLRP3.

### 3.3 Deletion of *ATG16L1* increases macrophage ROS accumulation by inhibiting autophagy

**FIGURE 3:**
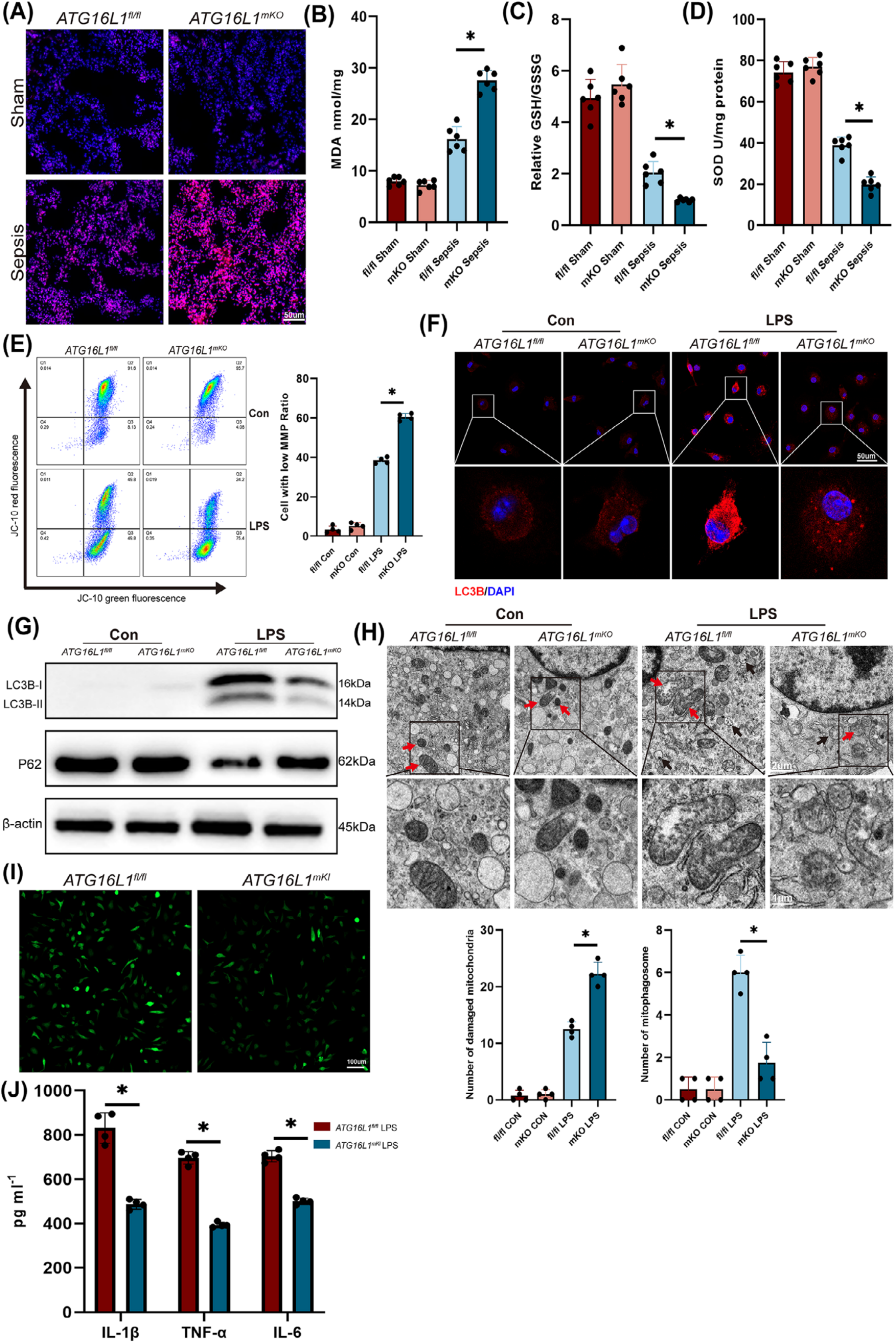
Deletion of ATG16L1 increases macrophage reactive oxygen species (ROS) accumulation by inhibiting autophagy. (A) Representative images of ROS fluorescence (red) detection in lung tissues. Blue indicates nuclei stained with DAPI (*n* = 6/group; scale bar: 50 µm). Levels of malondialdehyde (MDA) (B), GSH/GSSG (C) and superoxide dismutase (SOD) activity (D) in lung tissues from *ATG16L1^fl/fl^* and *ATG16L1^mKO^* mice treated with or without lipopolysaccharide (LPS) (*n* = 6/group). (E) Mitochondrial membrane potential detected by flow cytometry in *ATG16L1^fl/fl^* and *ATG16L1^mKO^* bone marrow‐derived macrophages (BMDMs) (*n* = 4/group). (F) Representative images of LC3B (red) immunofluorescence staining. Blue indicates nuclei stained with DAPI (*n* = 4/group; scale bar: 50 µm). (G) Western blot analysis of P62, LC3B‐I, LC3‐BII and β‐actin in BMDMs stimulated with LPS (*n* = 4/group). (H) Mitochondrial and phagosome ultrastructure observed using transmission electron microscopy in BMDMs with or without LPS treatment. Red arrows indicate mitochondria and black arrows indicate autophagosomes (*n* = 4/group; scale bar: 2 µm). (I) Representative immunofluorescence images of intracellular ROS in *ATG16L1^fl/fl^* and *ATG16L1^mKI^* BMDMs detected by DCFDA fluorescence probe (*n* = 4/group; scale bar: 100 µm). (J) Levels of inflammatory cytokines IL‐1β, TNF‐α and IL‐6 in the supernatants of *ATG16L1^fl/fl^* and *ATG16L1^mKI^* BMDMs (*n* = 4/group). Data are presented as the mean ± SEM. ^*^ *p* < .05.

ROS are crucial in activating the NLRP3 inflammasome.^24^ Disruption of mitochondrial homeostasis results in the generation of significant levels of ROS.^25^ Immunofluorescence revealed increased ROS in the lung tissues of *ATG16L1*‐deficient mice compared with *ATG16L1^fl/fl^* mice (Figure 3A). The findings revealed that myeloid *ATG16L1*‐deficient mice exhibited elevated MDA levels in the lung tissue and reduced levels of GSH/GSSG and SOD (Figure 3B‒D). Furthermore, in vitro, MMP significant decrease in *ATG16L1*‐deficient macrophages stimulated with LPS, which suggests mitochondrial damage and dysfunction (Figures 3E and S2A). Additionally, a significant increase in total cellular ROS and mitochondrial ROS levels was observed (Figure S2B‒D). Autophagy is crucial for clearing damaged mitochondria.^26^ When autophagy is defective, damaged mitochondria accumulate, hindering the scavenging of free radicals.^25^ Following LPS stimulation, normal BMDMs demonstrated autophagy activation, whereas *ATG16L1*‐deficient macrophages exhibited impaired LC3B lipidation and autophagy (Figure 3F,G). Electron microscopy revealed that *ATG16L1*‐deficient BMDMs accumulated damaged mitochondria and exhibited reduced autophagosome formation (Figure 3H). ROS regulatory factors, including FOXO3a, Nrf2 and P53, were examined. Following LPS treatment of BMDMs, the expression of FOXO3a and P53 was upregulated, and Nrf2 levels were downregulated. However, no significant differences were observed between BMDMs with or without *ATG16L1* deletion (Figure S3E). We hypothesised that the lack of noticeable differences in macrophages with *ATG16L1* deletion may be attributed to the complexity of the regulatory interactions between various signalling pathways. Furthermore, we overexpressed *ATG16L1* in BMDMs (Figure S1A). Following LPS stimulation, the production of ROS and of the NLRP3 signalling pathway in *ATG16L1^mKI^* BMDMs were reduced, and the secretion of IL‐1β, TNF‐α and IL‐6 in the BMDMs supernatant was also decreased (Figures 3I,J and S3F). 3‐MA, an autophagy inhibitor, abrogated the effects of ATG16L1 (Figure S3G,H). These results demonstrated that ATG16L1 regulates mitochondrial oxidative stress in macrophages and reduces ROS production through autophagy.

Mitotempo, a mitochondrial‐targeted ROS scavenger, notably decreases ROS levels in ALI (Figure S4A). By scavenging ROS, it significantly inhibits NLRP3 activation in lung tissues and BMDMs (Figure S4B). Furthermore, Mitotempo significantly alleviated lung tissues inflammation, reduced BALF cytokines and the inflammatory cytokine mRNA (Figure S4C‒F). In vitro, Mitotempo reduces ROS levels and prevents NLRP3 activation in LPS‐treated BMDMs, significantly decreased cytokines in the supernatant of LPS‐treated BMDMs and downregulated their mRNA expression (Figure S5A‒E). In summary, these results indicate that ROS accumulation in *ATG16L1*‐deficient macrophages mediates NLRP3 activation, thereby promoting macrophage inflammatory responses and exacerbating ALI.

### 3.4 *ATG16L1*‐deficient macrophages release inflammatory cytokines to damage alveolar epithelial cells after LPS treatment

**FIGURE 4:**
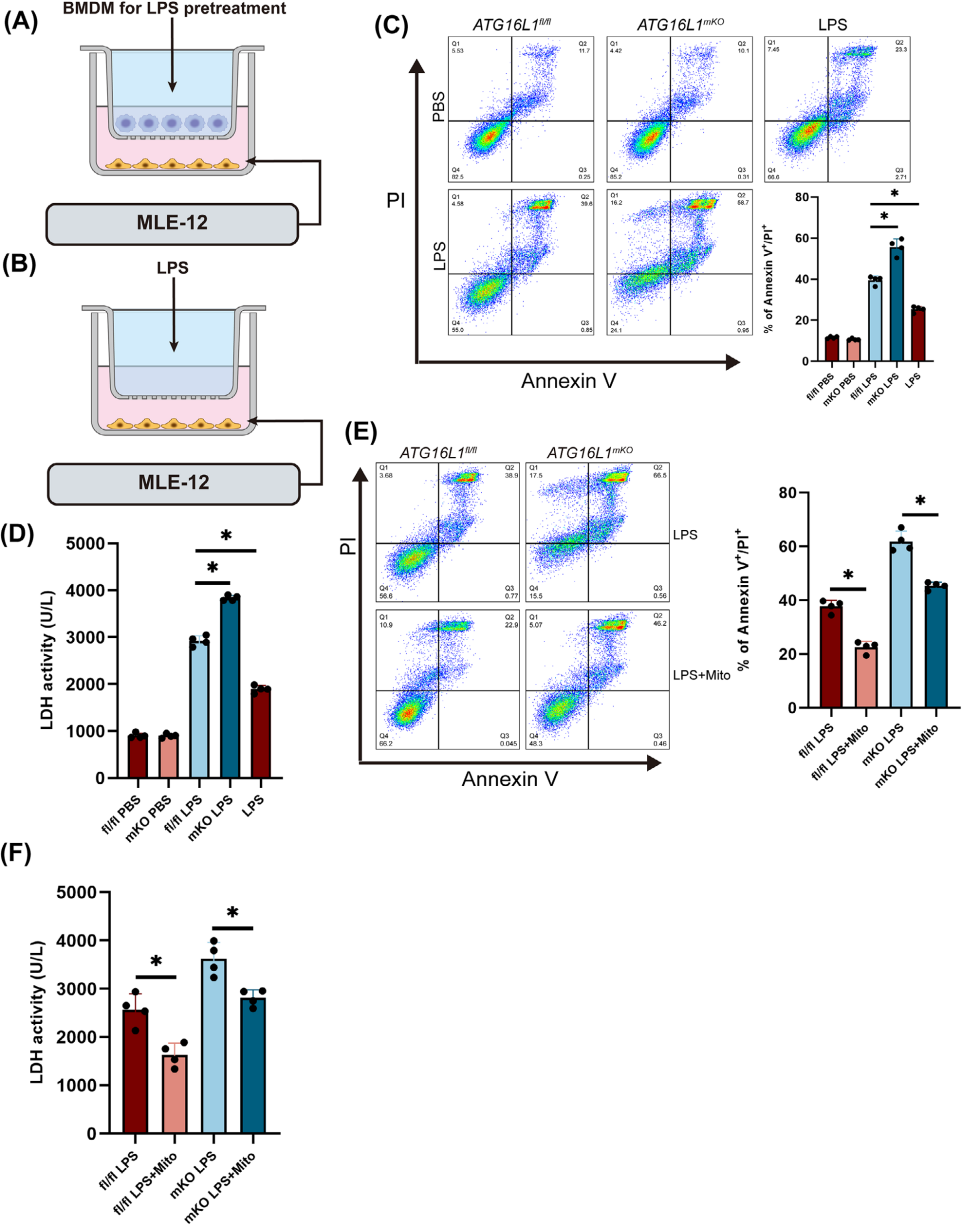
*ATG16L1*‐deficient macrophages release inflammatory cytokines to damage alveolar epithelial cells after lipopolysaccharide (LPS) treatment. Established a co‐culture system of bone marrow‐derived macrophages (BMDMs) and MLE‐12 cells with or without LPS treatment (A). LPS stimulation of MLE‐12 cells alone served as a control (B). (C) MLE‐12 cells extent of cell death measured by Annexin V and propidium iodide(PI) staining. (D) Levels of lactate dehydrogenase (LDH) in the supernatants of MLE‐12 cells. (E and F) MLE‐12 cells extent of cell measured by Annexin V and PI staining and levels of LDH in the supernatants cultured with *ATG16L1^fl/fl^* and *ATG16L1^mKO^* BMDMs with or without Mitotempo pretreatment (*n* = 4/group). Data are presented as the mean ± SEM. ^*^ *p* < .05.

Upon LPS stimulation, macrophages secrete inflammatory cytokines that mediate lung tissue damage.^27^ A co‐culture system of macrophages and alveolar epithelial cells was established to investigate the effects of inflammation‐activated macrophages on alveolar epithelial cells (Figure 4A). Considering that LPS in the co‐culture system could potentially affect the activity of alveolar epithelial cells, MLE‐12 cells were treated with LPS alone to exclude the impact of LPS on alveolar epithelial cells (Figure 4B). MLE‐12 cells co‐cultured with *ATG16L1‐*deficient BMDMs showed increased apoptosis and necrosis (Figure 4C). Additionally, elevated LDH levels in the supernatant of MLE‐12 cells indicated alveolar epithelial cell damage (Figure 4D). After Mitotempo treatment, reduced damage to alveolar epithelial cells was observed (Figure 4E,F). These results suggest that *ATG16L1*‐deficient macrophages exacerbate alveolar epithelial cell damage through an amplified inflammatory response, thereby aggravating tissue injury.

### 3.5 Inflammatory injury of alveolar epithelial cells enhances macrophage NLRP3 activation via double‐stranded DNA‒STING signalling

**FIGURE 5:**
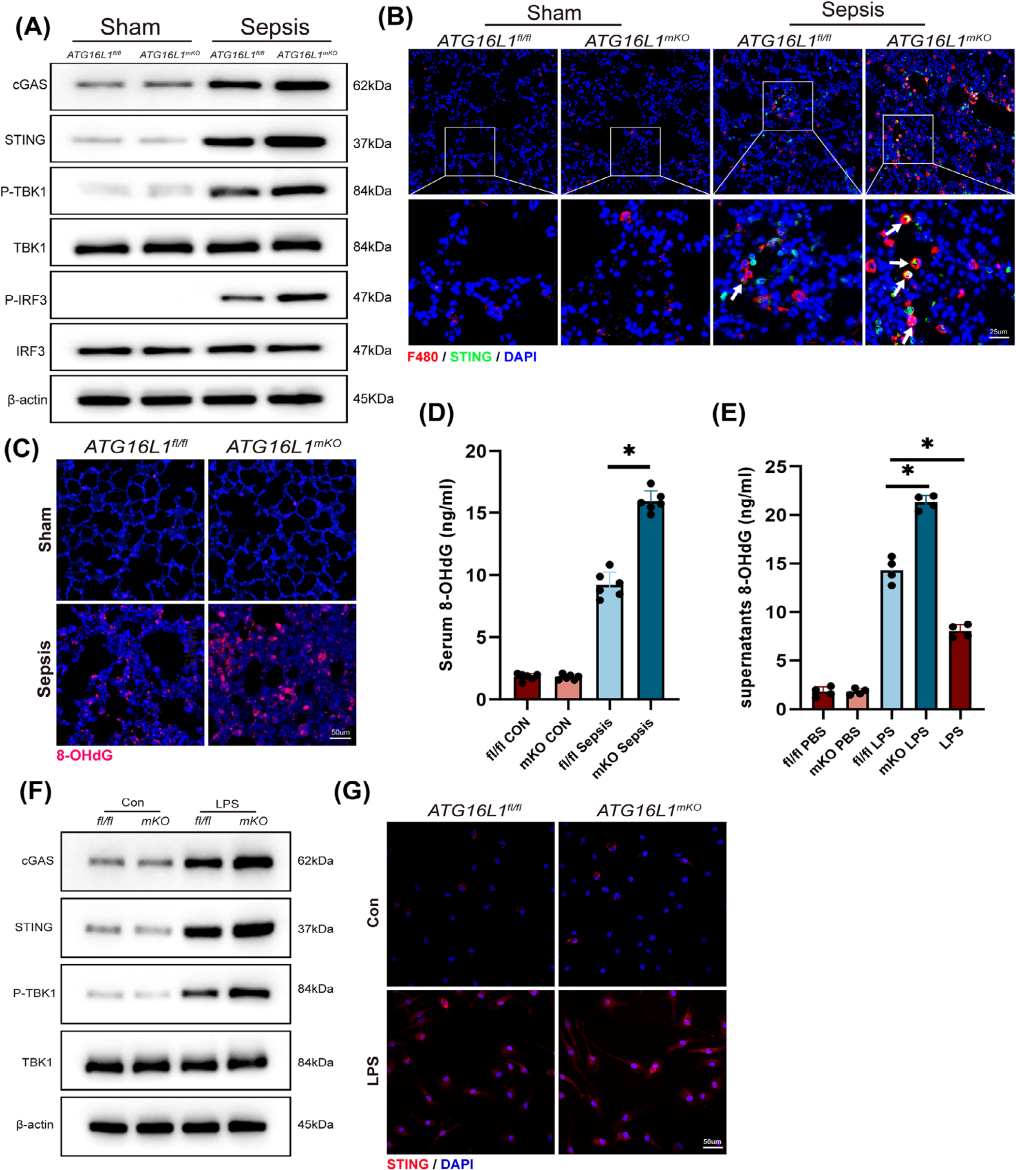
Inflammatory injury of alveolar epithelial cells enhances macrophage NLRP3 activation via dsDNA‒STING signalling. *ATG16L1^fl/fl^* and *ATG16L1^mKO^* mice were intraperitoneally injected with lipopolysaccharide (LPS; 10 mg/kg) or PBS. (A) Western blot analysis of cGAS, STING, P‐TBK1, TBK1, P‐IRF3, IRF3 and β‐actin in lung tissues (*n* = 6/group). (B) Representative immunofluorescence images of F4/80 (red) and STING (green) in lung tissues. Blue indicates nuclei stained with DAPI. White arrows indicate macrophages with elevated STING levels (*n* = 6/group; scale bar: 25 µm). (C and D) Representative immunofluorescence 8‐hydroxydeoxyguanosine (8‐OHdG) staining in *ATG16L1^fl/fl^* and *ATG16L1^mKO^* mice lung tissues (C) (scale bar: 50 µm) and serum 8‐OHdG levels (D) showing DNA damage (*n* = 6/group). (E) Levels of 8‐OHdG in the supernatants of MLE‐12 cells in the bone marrow‐derived macrophages (BMDMs) and MLE‐12 cells co‐culture system (*n* = 4/group). (F) Western blot analysis of cGAS, STING, P‐TBK1, TBK1 and β‐actin in BMDMs stimulated with MLE‐12 cells supernatants as conditioned media (*n* = 4/group). (G) Representative immunofluorescence images of STING (red) in BMDMs after stimulation with MLE‐12 cells conditioned media. Blue indicates nuclei stained with DAPI (*n* = 4/group; scale bar: 50 µm). Data are presented as the mean ± SEM. ^*^ *p* < .05.

Macrophage STING activation is critical in the development of ALI caused by sepsis.^28^, ^29^ We observed a significant activation of the cGAS‒STING of *ATG16L1*‐deficient mice lung following LPS stimulation (Figure 5A). Immunofluorescence co‐localisation revealed increased macrophage infiltration accompanied by STING activation in macrophages (Figure 5B). cGAS recognises dsDNA and facilitates the translocation of STING to the Golgi apparatus.^30^ The 8‐OHdG can be used to detect oxidative stress‐induced damage to nuclear and mitochondrial DNA. Levels of 8‐OHdG in the lung tissue and serum of *ATG16L1*‐deficient mice were markedly elevated after LPS treatment (Figure 5C,D). 8‐OHdG levels were significantly increased in the supernatant of MLE‐12 cells cultured with *ATG16L1^mKO^* BMDMs compared to *ATG16L1^fl/fl^* (Figure 5E). To explore the activation of macrophage STING by dsDNA released from alveolar epithelial cells. The supernatants of co‐cultured MLE‐12 cells were collected and used as conditioned medium (MLE‐12‐CM) to stimulate BMDMs, resulting in activation of STING in BMDMs (Figure 5F,G).

**FIGURE 6:**
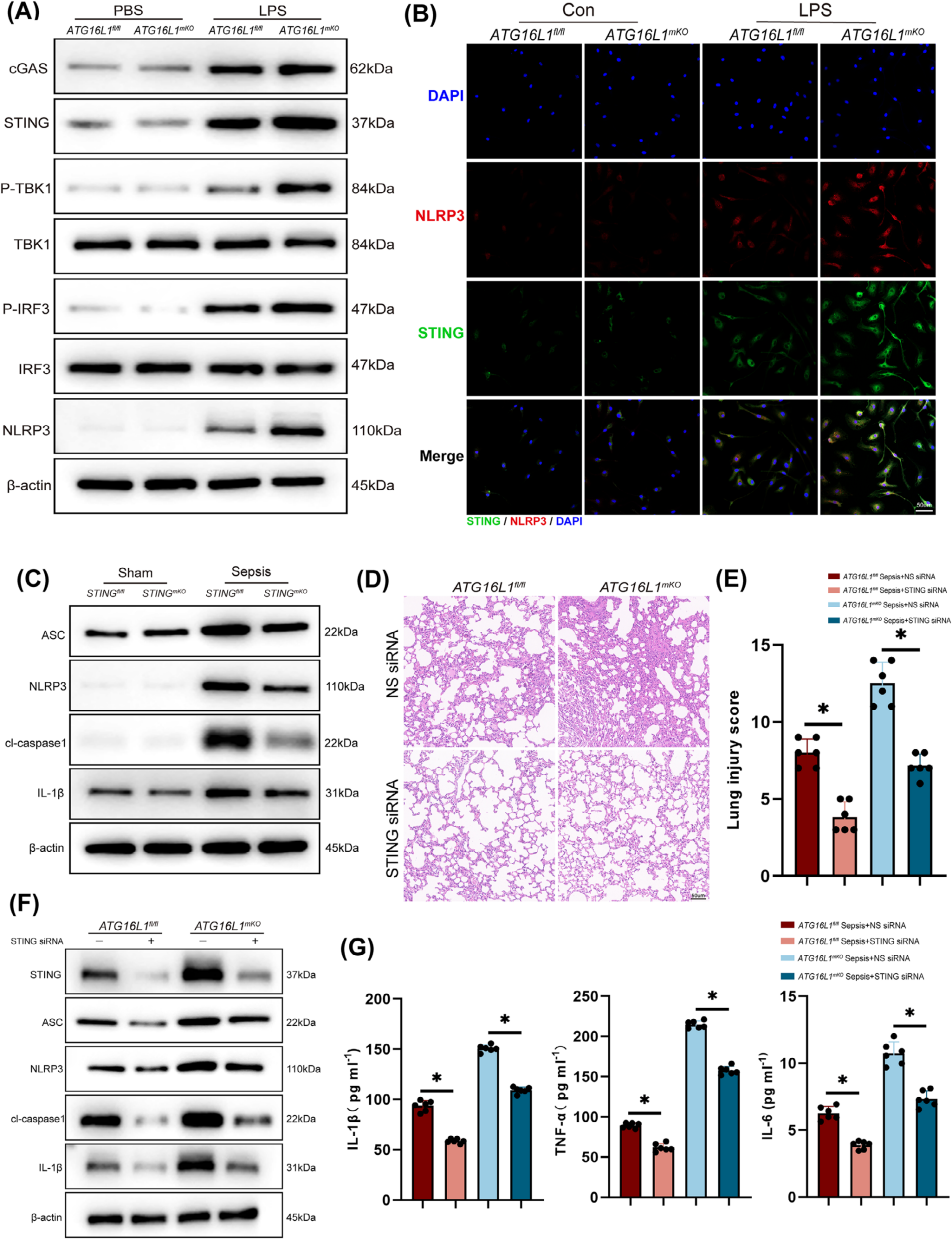
Activation of the STING signalling pathway promotes the activation of the NLRP3 inflammasome. (A and B) Stimulating bone marrow‐derived macrophages (BMDMs) with MLE‐12 cells supernatants as conditioned media. Western blot analysis of cGAS, STING, P‐TBK1, TBK1, P‐IRF3, IRF3, NLRP3 and β‐actin in BMDMs (A). Immunofluorescence staining showing activation and co‐localisation of STING (green) and NLRP3 (red). Blue indicates nuclei stained with DAPI (B) (*n* = 4/group; scale bar: 50 µm). (C) *STING^fl/fl^* and *STING^mKO^* mice were intraperitoneally injected with lipopolysaccharide (LPS) (10 mg/kg) or PBS. Lung tissues were collected after 24 h of LPS treatment. Representative Western blot analysis of ASC, NLRP3, cl‐caspase1, IL‐1β and β‐actin in lung tissues (*n* = 6/group). (D and E) *ATG16L1^fl/fl^* and *ATG16L1^mKO^* mice were subjected to intraperitoneal LPS (10 mg/kg) injection after in vivo transfection with STING siRNA or non‐specific siRNA (control). Haematoxylin and eosin (H&E) staining of lung tissue sections and lung tissues injury scores (*n* = 6/group; scale bar: 50 µm). (F and G) Western blot analysis of STING, ASC, NLRP3, cl‐caspase1, IL‐1β and β‐actin in lung tissues after in vivo transfection with STING siRNA (*n* = 6/group). Levels of inflammatory cytokines IL‐1β, TNF‐α and IL‐6 in BALF (*n* = 6/group). Data are presented as the mean ± SEM. ^*^ *p* < .05.

Previous researches have confirmed that the STING regulates the NLRP3 inflammasome activating.^31^, ^32^ Activation of the BMDMs STING‒NLRP3 was observed after MLE‐12 CM stimulation (Figure 6A,B). Subsequently, myeloid‐specific *STING*‐deficient mice were generated (Figure S1C), and reduced NLRP3 activation was observed in the lung tissue of *STING*‐deficient mice after sepsis induction (Figure 6C). BMDMs extracted from the bone marrow of *STING^fl/fl^* and *STING^mKO^* mice showed reduced NLRP3 activation upon LPS stimulation, as STING was depleted (Figure S6A). To further investigate the regulatory role of STING in the modulation of NLRP3, siRNA was constructed to knock down STING levels in mice and BMDMs. The experimental results demonstrated that in vivo knockdown of STING alleviated lung tissue inflammation, activation of the NLRP3 inflammasome pathway and reduced levels of inflammatory cytokines in BALF (Figure 6D‒G). In vitro experiments, STING siRNA effectively knocked down STING in BMDMs and reduced the activation of the NLRP3 inflammasome pathway and the secretion of inflammatory factors (Figure S6B‒D). Furthermore, C‐176, a targeted STING inhibitor, inhibited STING activation in macrophages and reduced NLRP3 activation (Figure S6E,F). BMDMs treated with C‐176 showed a significantly reduced production of inflammatory cytokines (Figure S6G,H). In conclusion, our experimental results indicate that *ATG16L1* deficiency leads to the accumulation of dsDNA in the lung tissue, subsequently activating the STING signalling pathway in macrophages. STING promotes NLRP3 activation, forming a positive feedback loop that amplifies the inflammatory response.

### 3.6 Clearance of dsDNA suppresses macrophage STING‒NLRP3 activation to protect lungs against septic injury

**FIGURE 7:**
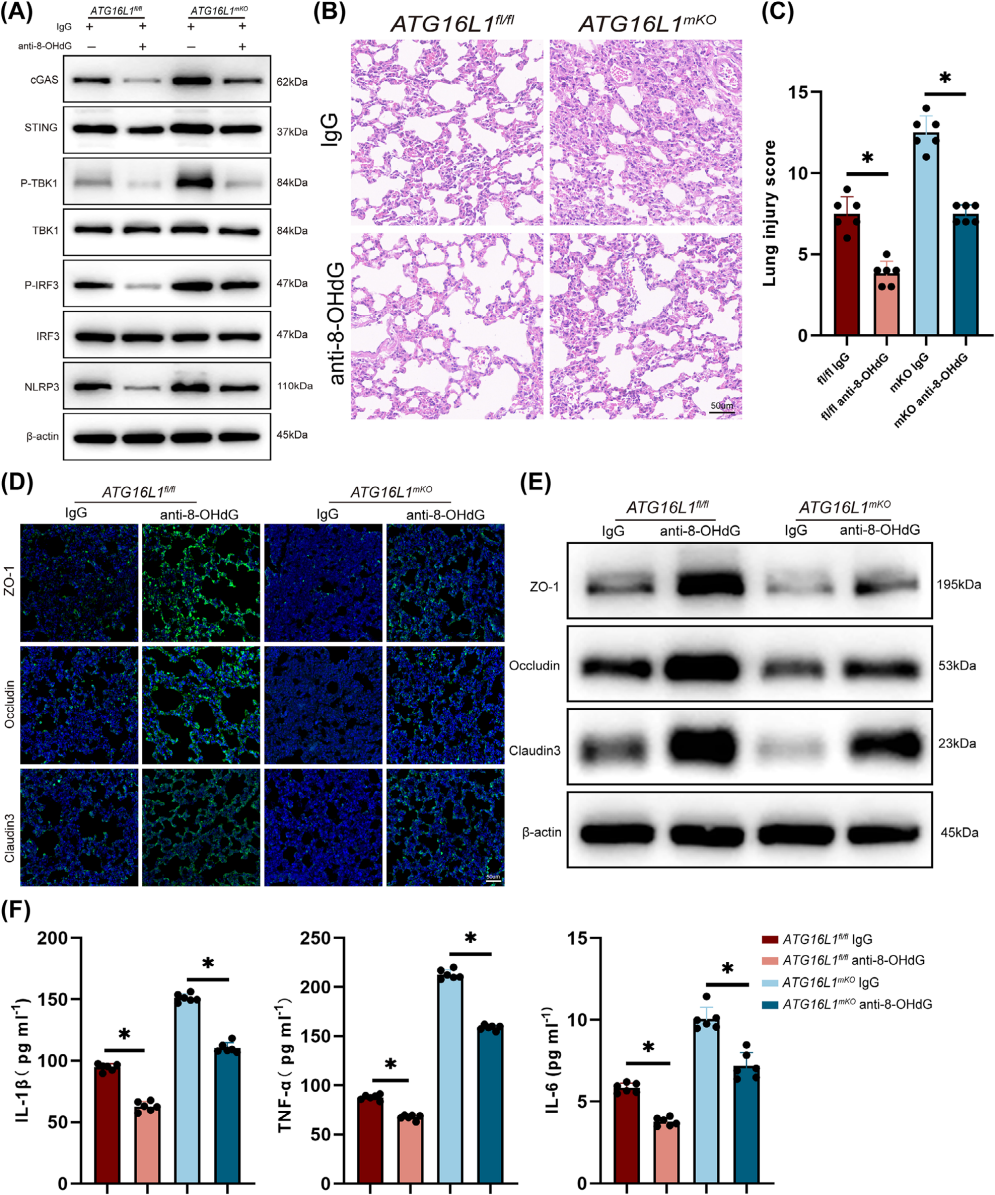
Clearance of double‐stranded DNA (dsDNA) suppresses macrophage STING‒NLRP3 activation to protect lungs against septic injury. Immunoglobulin G (IgG) or anti‐8‐OHG antibody were pretreated 12 h before lipopolysaccharide (LPS)‐induced sepsis modelling. Lung tissues and BALF were collected after 24 h of LPS treatment. (A) Western blot analysis of cGAS, STING, P‐TBK1, TBK1, P‐IRF3, IRF3, NLRP3 and β‐actin in lung tissues (*n* = 6/group). (B and C) Haematoxylin and eosin (H&E) staining of lung tissue sections and lung tissues injury scores (*n* = 6/group; scale bar: 50 µm). (D) Representative immunofluorescence images of ZO‐1 (green), Occludin (green) and Claudin 3 (green) in lung tissues. Blue indicates nuclei stained with DAPI (*n* = 6/group; scale bar: 50 µm). (E) Western blot analysis of ZO‐1, Occludin, Claudin 3 and β‐actin in lung tissues (*n* = 6/group). (F) Levels of inflammatory cytokines IL‐1β, TNF‐α and IL‐6 in BALF (*n* = 6/group). Data are presented as the mean ± SEM. ^*^ *p* < .05.

To explore whether blocking the STING‒NLRP3 pathway would be beneficial for controlling lung inflammation, mice with sepsis were pretreated with anti‐8‐OHdG antibodies. Our results showed that anti‐8‐OHdG antibody treatment reduced STING‒NLRP3 activation in the lung tissue (Figure 7A). Similarly, anti‐8‐OHdG treatment of BMDMs decreased STING‒NLRP3 activation (Figure S7A). Additionally, anti‐8‐OHdG treatment significantly alleviated lung tissue inflammation (Figure 7B,C). ZO‐1, Occludin and Claudin 3 levels in lung tissues were upregulated after anti‐8‐OHdG treatment, indicating an improvement in lung barrier structure (Figure 7D,E). Furthermore, the concentrations of inflammatory factors in BALF significantly reduced after clearing dsDNA (Figures 7F and S7B). In summary, our study suggests that clearing damaged DNA in vivo can reduce lung tissues inflammation.

### 3.7 Myeloid‐ATG16L1 overexpression alleviates septic lung injury

**FIGURE 8:**
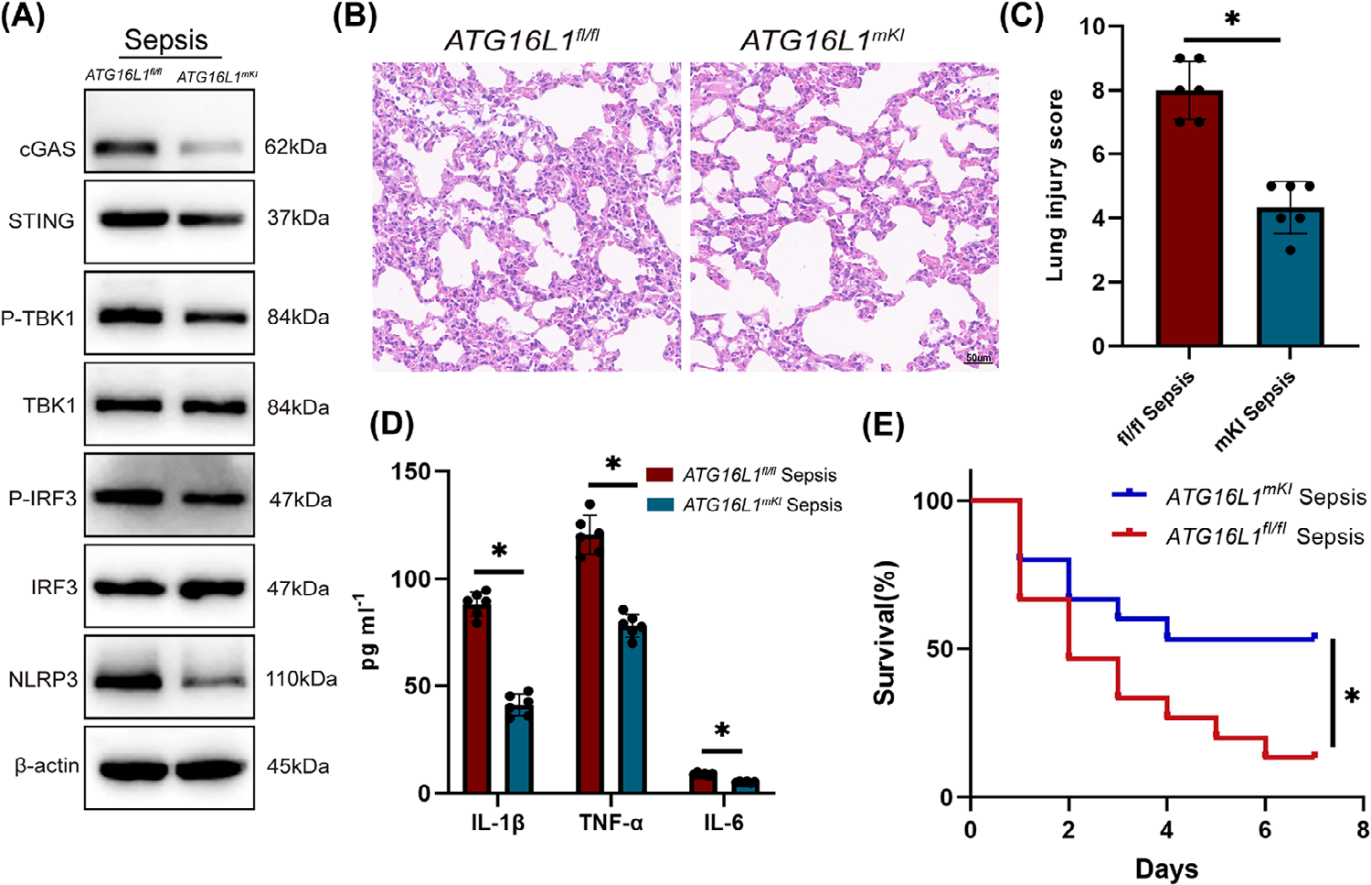
Myeloid‐ATG16L1 overexpression alleviates septic lung injury. *ATG16L1^fl/fl^* and *ATG16L1^mKI^* mice were intraperitoneally injected with lipopolysaccharide (LPS) (10 mg/kg) or PBS. Lung samples and BALF were collected 24 h after the treatment. (A) Western blot analysis of cGAS, STING, P‐TBK1, TBK1, P‐IRF3, IRF3, NLRP3 and β‐actin proteins in lung tissues (*n* = 6/group). (B and C) Haematoxylin and eosin (H&E) staining of lung tissue sections and lung tissues injury scores (*n* = 6/group; scale bar: 50 µm). (D) Levels of inflammatory cytokines IL‐1β, TNF‐α and IL‐6 in the BALF (*n* = 6/group). (E) Survival analysis of *ATG16L1^fl/fl^* and *ATG16L1^mKI^* mice after LPS (20 mg/kg) treatment (*n* = 15/group). Data are presented as the mean ± SEM. ^*^ *p* < .05.

To verify the protective role of ATG16L1 against sepsis‐induced lung injury, *ATG16L1* overexpression mice were generated (Figure S1A). Compared to *ATG16L1^fl/fl^* mice, the overexpression of ATG16L1 notably inhibited the cGAS‒STING‒NLRP3 signalling pathway in lung tissue subjected to LPS treatment (Figure 8A). Additionally, *ATG16L1* overexpression markedly reduced lung inflammation in septic mice (Figure 8B‒D), leading to an improved 7‐day survival rate (Figure 8E). In summary, *ATG16L1* overexpression provides significant benefits against sepsis‐induced lung injury.

## 4 DISCUSSION

An analysis of 30 606 inflammatory bowel disease (IBD) patients revealed that the *ATG16L1* variant rs2241880 is a persistent risk factor for the development of IBD and may influence patient prognosis.^33^ Autophagy mediated by ATG16L1 in microglia ameliorates central nervous system inflammation and alleviates inflammatory responses and clinical symptoms of experimental autoimmune encephalomyelitis.^34^ Nevertheless, the function of macrophage ATG16L1 in regulating lung inflammation and the NLRP3 inflammasome activating remains to be elucidated. Here, we found that myeloid *ATG16L1*‐knockout mice were observed to exhibit exacerbated lung inflammation, reduced survival rates and increased activation of the NLRP3 inflammasome. Further investigation indicated that loss of ATG16L1 impaired autophagy and activated the NLRP3 inflammasome by accumulating of mitochondrial ROS, which triggered macrophage inflammatory activation. Secretion of inflammatory cytokines by activated macrophages damaged alveolar epithelial cells and releases dsDNA. The dsDNA released by damaged alveolar epithelial cells activates the STING‒NLRP3 pathway, thereby creating a positive feedback loop for NLRP3 activation. The positive feedback activation of NLRP3 triggers an uncontrollable inflammatory response, thereby exacerbating sepsis‐induced ALI.

The immune system is pivotal in the onset of sepsis. Macrophages, which are the predominant immune cells in lung tissue, preserving lung homeostasis and protecting against pathogenic invasion.^35^ During ALI, macrophages and their secreted exosomes activate neutrophils and promote their secretion of inflammatory factors, thereby exacerbating ALI.^36^ TNF‐α, released by macrophage cells, induces Granulocyte‐macrophage colony‐stimulating factor (GM‐CSF) expression in the alveolar epithelium. GM‐CSF maintains the alveolar barrier function by promoting the proliferation of alveolar epithelial cells.^37^ The NLRP3 inflammasome is an important sensors in the innate immune and expressed in myeloid immune cells.^38^ In COVID‐19 patients, the activation of inflammasomes in lung macrophages leads to the release of IL‐1 and IL‐18, which may cause excessive lung inflammation.^39^ However, the released inflammatory factors may also aid in combating viral invasion. LPS treatment activates the NLRP3 inflammasome in alveolar macrophages, mediates inflammatory response, and plays a significant role in ALI.^11^, ^40^ Potassium efflux, increased cytosolic calcium levels, and ROS accumulation effectively activate the NLRP3 inflammasome.^24^ ROS are one of the earliest discovered activators of the NLRP3 inflammasome. ROS production promotes the relocation of NLRP3 to the mitochondria‐associated ER membrane and recruits ASC, thereby facilitating NLRP3 inflammasome activation.^41^ Our study confirmed that upon LPS stimulation of *ATG16L1*‐deficient macrophages, cytosolic ROS levels significantly increased and the assembly of the NLRP3 inflammasome was enhanced. Scavenging ROS effectively prevents NLRP3 inflammasome activation and mitigates inflammatory damage in lung tissue.

The balance between ROS production and elimination is crucial to avoid cellular damage and maintain homeostasis.^42^ FOXO3a, Nrf2 and P53 play a crucial role in regulating ROS production. Mitochondrial ROS can activate Nrf2, whereas excessive ROS suppresses Nrf2.^43^ At physiological levels, P53 transactivates antioxidant genes to control ROS levels, whereas at elevated levels, P53 facilitates ROS generation.^44^ In adipocytes deficient in autophagy proteins ATG3 and ATG16L1, the accumulation of dysfunctional mitochondria increases, resulting in increased activation of Nrf2 and Keap1.^45^ In *ATG5*‐knockout mice with impaired autophagy, P62 accumulation increases and Nrf2 activation plays a critical role in APAP‐induced acute liver injury.^46^ Mitochondria are the most important and typical sources of ROS in macrophages during antibacterial responses.^47^ When mitochondrial homeostasis is disrupted, mitochondria generate and release large amounts of ROS, ultimately activating NLRP3 inflammasome.^48^ Following the LPS stimulation of macrophages, mitophagy plays a major role in ROS elimination, inflammation inhibition and pyroptosis suppression.^11^ ROS activates autophagy, which degrades and recycles damaged intracellular macromolecules and dysfunctional organelles.^49^ ATG16L1, a key molecule that promotes LC3 lipidation, has become the focus of IBD research in recent years.^50^ After Salmonella enterica serovar Typhimurium infection in the small intestine and cecum, ATG16L1‐mediated autophagy promotes Salmonella clearance, thereby reducing intestinal inflammation.^51^ ATG16L1 also prevents necroptosis in intestinal epithelial cells. *ATG16L1* deficiency disrupts mitochondrial homeostasis and enhances endoplasmic reticulum stress.^52^ LPS stimulation of *ATG16L1*‐deficient macrophages induces the production of substantial amounts of the inflammatory cytokines.^18^ Furthermore, autophagy regulates activation of inflammasomes, and defects in autophagy lead to impaired degradation of NLRP3.^53^ However, the regulatory mechanism of ATG16L1 on NLRP3 and its role in sepsis remain unclear. Our study confirmed that upon LPS stimulation of *ATG16L1*‐deficient macrophages, cytosolic ROS levels significantly increased, thereby promoting NLRP3 inflammasome activation. These results underscore the role of ATG16L1‐mediated autophagy in the regulation of the NLRP3 inflammasome.

As a DAMP, DNA has strong immunogenicity. The cGAS‒STING is the primary pathway recognised intracellular DNA recognition.^54^ Upon binding of dsDNA to cGAS, the catalytic activity of cGAS is activated through an allosteric mechanism, resulting in the production of 2,3‐cGAMP, which subsequently activates the downstream STING pathway. STING activation triggers TANK‐binding kinase 1 (TBK1) and induces the transcription of interferon‐stimulated genes involved in antibacterial and antitumor responses.^55^, ^56^, ^57^ Activated neutrophils release neutrophil extracellular traps, which activate the pulmonary cGAS‒STING pathway, thereby exacerbating LPS‐induced lung inflammation.^58^ In the later stages, STING is transported to the lysosome for degradation, with the blockade of STING degradation playing an important role in anti‐tumour responses.^59^ In dendritic cells and fibroblasts, autophagy involving ATG5, ATG9, ATG12 and ATG16L1 contributes to STING degradation.^60^ Furthermore, cGAS‒STING can activate autophagy independently of TBK1 activation and the INF mechanism.^61^ STING promotes lysosomal activity through the activation of transcription factor EB, thereby resisting pathogen invasion.^62^ Mechanistically, STING activation directly promotes the binding of lipidated LC3B to the autophagosome membrane via the WD40 domain of ATG16L.^63^ This suggests that the interaction between ATG16L1 and STING may influence various diseases, including inflammation, cancer and Alzheimer's disease.

Our previous study demonstrated that STING‐mediated NLRP3 activation aggravated liver ischaemia‒reperfusion injury in ageing macrophages.^31^ Loss of myeloid XBP1 impairs BNIP3‐mediated mitophagy and promotes macrophage cGAS/STING/NLRP3 activation through leakage of mtDNA into the cytosol, thereby exacerbating liver fibrosis.^64^ In sepsis‐induced ALI, LPS increased the transcription factor c‐Myc, which activated the STING‒NLRP3.^28^ Our previous research found that the knockout of *ATG16L1* inhibits lipophagy in macrophages, having a key role in the progression of metabolic dysfunction‐associated steatohepatitis.^65^ In this context, activated macrophages were observed to interact with alveolar epithelial cells, thereby triggering the STING‒NLRP3 axis, resulting in excessive activation of NLRP3 and causing an uncontrollable inflammatory response.

Several drugs target ATG16L1/NLRP3/STING and play a crucial role in controlling inflammatory responses. Tocilizumab and Ustekinumab significantly upregulates ATG16L1 levels, alleviating intestinal inflammation.^66^ Norwogonin inhibits the NLRP3/nuclear factor‐κB pathway, thereby decreasing the release of inflammatory factors and alleviating LPS‐induced ALI.^67^ Clioquinol directly inhibits NLRP3 inflammasome activation, reduces macrophage inflammatory factor production, and alleviates experimental sepsis, colitis and acute peritonitis.^68^ Gelsevirine and Glycyrrhiza uralensis improve sepsis by inhibiting the cGAS‒STING signalling pathway in microglia and macrophages.^69^, ^70^

In summary, this study revealed that ATG16L1 regulates macrophage NLRP3 activation during sepsis and interacts with alveolar epithelial cells to form a positive feedback activation loop during sepsis‐induced lung injury. However, this study has certain limitations. Owing to the lack of clinical samples, the role and mechanisms of ATG16L1 in humans require further investigation. Furthermore, it remains to be explored whether ATG16L1‐mediated autophagy influences the clearance of dsDNA derived from alveolar epithelial cells.

## AUTHOR CONTRIBUTIONS

Yan Bai, Xinyu Zhan and Qing Zhu performed experiments. Xingyue Ji, Yingying Lu, Yiyun Gao, Fei Li and Zhu Guan analysed the data. Haoming Zhou and Zhuqing Rao designed the research and wrote the manuscript. All authors reviewed the manuscript.

## CONFLICT OF INTEREST STATEMENT

The authors declare they have no conflicts of interest.

## ETHICS STATEMENT

Animal procedures complied with the legal and ethical guidelines set forth in the protocol approved by the Institutional Animal Care and Use Committee of Nanjing Medical University (IACUC‐2402019).

## Supporting information




**FIGURE S1** Myeloid‐specific ATG16L1 deficiency exacerbates sepsis‐induced lung injury. (A‒C) Western blot analysis of ATG16L1 (A), NLRP3 (B), STING (C) and β‐actin proteins in bone marrow‐derived macrophages (BMDMs). (D) Semiquantitative analysis of lung tissues was performed based on lung injury scores (*n* = 6/group). (E) Relative expression of *IL‐1β*, *TNF‐α* and *IL‐6* mRNA in lung tissues detected by RT‐qPCR (*n* = 6/group). Data are presented as the mean ± SEM. ^*^ *p* < .05.

**FIGURE S2** ATG16L1 deficiency promotes macrophage NLRP3 inflammasome activation to aggravate acute lung injury (ALI). (A) Concentration of inflammatory cytokines IL‐1β, TNF‐α and IL‐6 in *NLRP3^fl/fl^* and *NLRP3^mKO^* murine BALF (*n* = 6/group). (B) Western blot analysis of ASC, NLRP3, cl‐caspase1, IL‐1β and β‐actin in lipopolysaccharide (LPS)‐treated *ATG16L1^fl/fl^* and *ATG16L1^mKO^* bone marrow‐derived macrophages (BMDMs) (*n* = 4/group). (C) Relative mRNA expression levels of *IL‐1β*, *TNF‐α* and *IL‐6* in *ATG16L1^fl/fl^* and *ATG16L1^mKO^* BMDMs (*n* = 4/group). (D) Before treating *ATG16L1^fl/fl^* and *ATG16L1^mKO^* BMDMs with LPS, pretreated with the NLRP3 inhibitor MCC950 (10 µM) for 6 h. Western blot images of NLRP3 and β‐actin in BMDMs (*n* = 4/group). (E) Levels of inflammatory cytokines IL‐1β, TNF‐α and IL‐6 in the supernatants of BMDMs with MCC950 treatment. (F and G) MCC950 (10 mg/kg) was administered 12 h before LPS‐induced *ATG16L1^fl/fl^* and *ATG16L1^mKO^* mice sepsis modelling. Haematoxylin and eosin (H&E) staining of lung tissue sections and lung tissues injury scores (*n* = 6/group; scale bar: 50 µm). Data are presented as the mean ± SEM. ^*^ *p* < .05.

**FIGURE S3** Deletion of ATG16L1 increases macrophage reactive oxygen species (ROS) accumulation by inhibiting autophagy. (A) Immunofluorescence detection of mitochondrial membrane potential in *ATG16L1^fl/fl^* and *ATG16L1^mKO^* bone marrow‐derived macrophages (BMDMs) (*n* = 4/group; scale bar: 200 µm). (B and C) Levels of intracellular ROS in *ATG16L1^fl/fl^* and *ATG16L1^mKO^* BMDMs detected by DCFDA fluorescence probe. Representative immunofluorescence images of ROS (B) (scale bar: 100 µm). Representative flow cytometry plot of ROS (C). (D) Mitochondrial reactive oxygen species levels detected by MitoSOX Red fluorescence probe (*n* = 4/group; scale bar: 100 µm). (E) Western blot analysis of FOXOa3, Nrf2, P53 and β‐actin in *ATG16L1^fl/fl^* and *ATG16L1^mKO^* BMDMs (*n* = 4/group). (F) Western blot analysis of ASC, NLRP3, cl‐caspase1, IL‐1β and β‐actin in lipopolysaccharide (LPS)‐treated *ATG16L1^fl/fl^* and *ATG16L1^mKI^* BMDMs (*n* = 4/group). (G) Representative fluorescence images of ROS in *ATG16L1^fl/fl^* and *ATG16L1^mKI^* BMDMs treated with 3‐MA (*n* = 4/group). (H) Mitochondrial membrane potential detected by flow cytometry in *ATG16L1^fl/fl^* and *ATG16L1^mKI^* BMDMs treated with 3‐MA (*n* = 4/group). Data are presented as the mean ± SEM. ^*^ *p* < .05.

**FIGURE S4** Clearance of reactive oxygen species (ROS) inhibits the activation of NLRP3 and improves lung tissues inflammation. (A) Representative images of ROS fluorescence assay in lung tissue using the DHE fluorescent probe (red) in *ATG16L1^fl/fl^* and *ATG16L1^mKO^* septic mice, with or without Mitotempo pretreatment. Blue indicates nuclei stained with DAPI (*n* = 6/group; scale bar: 50 µm). (B) Western blot analysis of ASC, NLRP3, cl‐caspase1, IL‐1β and β‐actin in ATG16L1*^fl/fl^* and ATG16L1*^mKO^* septic mice lung tissues treated with Mitotempo (*n* = 6/group). (C and D) Haematoxylin and eosin (H&E) staining of lung tissue sections and lung tissues injury scores (*n* = 6/group; scale bar: 50 µm). (E) Levels of inflammatory cytokines IL‐1β, TNF‐α and IL‐6 in ATG16L1*^fl/fl^* and ATG16L1*^mKO^* septic mice BALF (*n* = 6/group). (F) Relative expression of *IL‐1β*, *TNF‐α* and *IL‐6* mRNA in lung tissues treated with Mitotempo (*n* = 6/group). Data are presented as the mean ± SEM. ^*^ *p* < .05.

**FIGURE S5** Clearance of reactive oxygen species (ROS) suppresses bone marrow‐derived macrophages (BMDMs) inflammatory activation. (A) Intracellular ROS levels detected by flow cytometry in *ATG16L1^fl/fl^* and *ATG16L1^mKO^* BMDMs Mitotempo pretreatment (*n* = 4/group). (B) Representative immunofluorescence images of ROS detected by DCFDA fluorescence probe in *ATG16L1^fl/fl^* and *ATG16L1^mKO^* BMDMs with Mitotempo treatment (*n* = 4/group; scale bar: 100 µm). (C) Western blot analysis of ASC, NLRP3, cl‐caspase1, IL‐1β and β‐actin in ATG16L1*^fl/fl^* and ATG16L1*^mKO^* BMDMs treated with Mitotempo (*n* = 4/group). (D) Concentration of inflammatory cytokines IL‐1β, TNF‐α and IL‐6 in the supernatants of BMDMs with Mitotempo treatment (*n* = 4/group). (E) Relative expression of *IL‐1β*, *TNF‐α* and *IL‐6* mRNA in BMDMs treated with Mitotempo (*n* = 4/group). Data are presented as the mean ± SEM. ^*^ *p* < .05.

**FIGURE S6** Activation of the STING signalling pathway promotes the activation of the NLRP3 inflammasome. (A) Bone marrow‐derived macrophages (BMDMs) were isolated from *STING^fl/fl^* and *STING^mKO^* mice. Representative Western blot analysis of ASC, NLRP3, cl‐caspase1, IL‐1β and β‐actin in BMDMs after lipopolysaccharide (LPS) stimulation. (B) BMDMs were transfected with STING siRNA or non‐specific siRNA (control) and then stimulated with 100 ng/mL of LPS. Protein levels of STING, ASC, NLRP3, cl‐caspase1, IL‐1β and β‐actin were measured using Western blotting (*n* = 4/group). (C and D) Levels of inflammatory cytokines IL‐1β, TNF‐α and IL‐6 in the supernatants of BMDMs transfected with STING siRNA (*n* = 4/group). (E) Western blot analysis of STING, P‐TBK1, NLRP3, cl‐caspase1 and β‐actin in *ATG16L1^fl/fl^* and *ATG16L1^mKO^* BMDMs pretreated with the STING inhibitor C‐176 (*n* = 4/group). (F) Immunofluorescence staining showed the activation and co‐localisation of NLRP3 (red) and STING (green) after pretreatment with STING inhibitor C‐176. Blue indicates nuclei stained with DAPI (*n* = 4/group, scale bar: 50 µm). (G and H) Levels of inflammatory cytokines IL‐1β, TNF‐α and IL‐6 in the supernatants of BMDMs pretreated with the STING inhibitor C‐176 (*n* = 4/group). Data are presented as the mean ± SEM. ^*^ *p* < .05.

**FIGURE S7** Clearance of double‐stranded DNA (dsDNA) suppresses macrophage STING‒NLRP3 activation to protect lungs against septic injury. (A) Western blot analysis of cGAS, STING, P‐TBK1, TBK1, P‐IRF3, IRF3, NLRP3 and β‐actin in bone marrow‐derived macrophages (BMDMs) after treatment with anti‐8‐OHG antibody (*n* = 4/group). (B) Relative mRNA expression levels of *IL‐1β*, *TNF‐α* and *IL‐6* in lung tissues after treatment with anti‐8‐OHG antibody (*n* = 6/group). Data are presented as the mean ± SEM. ^*^ *p* < .05.

Supporting Information

## Data Availability

All data supporting the findings of this study are available within the paper.

## References

[1] van der Poll T , van de Veerdonk FL , Scicluna BP , Netea MG . The immunopathology of sepsis and potential therapeutic targets. Nat Rev Immunol. 2017;17(7):407‐420. doi:10.1038/nri.2017.36 28436424

[2] Rudd KE , Johnson SC , Agesa KM , et al. Global, regional, and national sepsis incidence and mortality, 1990–2017: analysis for the Global Burden of Disease Study. Lancet. 2020;395(10219):200‐211. doi:10.1016/s0140-6736(19)32989-7 31954465 PMC6970225

[3] Martin GS , Mannino DM , Eaton S , Moss M . The epidemiology of sepsis in the United States from 1979 through 2000. N Engl J Med. 2003;348(16):1546‐1554. doi:10.1056/NEJMoa022139 12700374

[4] Aziz M , Ode Y , Zhou M , et al. B‐1a cells protect mice from sepsis‐induced acute lung injury. Mol Med. 2018;24(1):26. doi:10.1186/s10020-018-0029-2 30134811 PMC6016888

[5] Singer M , Deutschman CS , Seymour CW , et al. The Third International Consensus Definitions for Sepsis and Septic Shock (Sepsis‐3). JAMA. 2016;315(8):801‐810. doi:10.1001/jama.2016.0287 26903338 PMC4968574

[6] Nedeva C . Inflammation and cell death of the innate and adaptive immune system during sepsis. Biomolecules. 2021;11(7):1011. doi:10.3390/biom11071011 34356636 PMC8301842

[7] van der Poll T , Shankar‐Hari M , Wiersinga WJ . The immunology of sepsis. Immunity. 2021;54(11):2450‐2464. doi:10.1016/j.immuni.2021.10.012 34758337

[8] Mikkelsen ME , Shah CV , Meyer NJ , et al. The epidemiology of acute respiratory distress syndrome in patients presenting to the emergency department with severe sepsis. Shock. 2013;40(5):375‐381. doi:10.1097/SHK.0b013e3182a64682 23903852 PMC3800497

[9] Magupalli VG , Negro R , Tian Y , et al. HDAC6 mediates an aggresome‐like mechanism for NLRP3 and pyrin inflammasome activation. Science. 2020;369(6510):eaas8995. doi:10.1126/science.aas8995 32943500 PMC7814939

[10] Zhong WJ , Liu T , Yang HH , et al. TREM‐1 governs NLRP3 inflammasome activation of macrophages by firing up glycolysis in acute lung injury. Int J Biol Sci. 2023;19(1):242‐257. doi:10.7150/ijbs.77304 36594089 PMC9760435

[11] Wu D , Zhang H , Wu Q , et al. Sestrin 2 protects against LPS‐induced acute lung injury by inducing mitophagy in alveolar macrophages. Life Sci. 2021;267:118941. doi:10.1016/j.lfs.2020.118941 33359748

[12] Levine B , Mizushima N , Virgin HW . Autophagy in immunity and inflammation. Nature. 2011;469(7330):323‐335. doi:10.1038/nature09782 21248839 PMC3131688

[13] Deretic V , Saitoh T , Akira S . Autophagy in infection, inflammation and immunity. Nat Rev Immunol. 2013;13(10):722‐737. doi:10.1038/nri3532 24064518 PMC5340150

[14] Pu Q , Gan C , Li R , et al. Atg7 deficiency intensifies inflammasome activation and pyroptosis in pseudomonas sepsis. J Immunol. 2017;198(8):3205‐3213. doi:10.4049/jimmunol.1601196 28258192 PMC5382979

[15] Otomo C , Metlagel Z , Takaesu G , Otomo T . Structure of the human ATG12∼ATG5 conjugate required for LC3 lipidation in autophagy. Nat Struct Mol Biol. 2013;20(1):59‐66. doi:10.1038/nsmb.2431 23202584 PMC3540207

[16] Gong X , Wang Y , Tang Y , et al. ATG16L1 adopts a dual‐binding site mode to interact with WIPI2b in autophagy. Sci Adv. 2023;9(9):eadf0824. doi:10.1126/sciadv.adf0824 36857448 PMC9977175

[17] Magné J , Green DR . LC3‐associated endocytosis and the functions of Rubicon and ATG16L1. Sci Adv. 2022;8(43):eabo5600. doi:10.1126/sciadv.abo5600 36288306 PMC9604520

[18] Saitoh T , Fujita N , Jang MH , et al. Loss of the autophagy protein Atg16L1 enhances endotoxin‐induced IL‐1beta production. Nature. 2008;456(7219):264‐268. doi:10.1038/nature07383 18849965

[19] Aden K , Tran F , Ito G , et al. ATG16L1 orchestrates interleukin‐22 signaling in the intestinal epithelium via cGAS‒STING. J Exp Med. 2018;215(11):2868‐2886. doi:10.1084/jem.20171029 30254094 PMC6219748

[20] Zhong W , Rao Z , Xu J , et al. Defective mitophagy in aged macrophages promotes mitochondrial DNA cytosolic leakage to activate STING signaling during liver sterile inflammation. Aging Cell. 2022;21(6):e13622. doi:10.1111/acel.13622 35599014 PMC9197407

[21] Yang HH , Duan JX , Liu SK , et al. A COX‐2/sEH dual inhibitor PTUPB alleviates lipopolysaccharide‐induced acute lung injury in mice by inhibiting NLRP3 inflammasome activation. Theranostics. 2020;10(11):4749‐4761. doi:10.7150/thno.43108 32308747 PMC7163435

[22] Guey B , Bodnar M , Manié SN , Tardivel A , Petrilli V . Caspase‐1 autoproteolysis is differentially required for NLRP1b and NLRP3 inflammasome function. Proc Natl Acad Sci U S A. 2014;111(48):17254‐17259. doi:10.1073/pnas.1415756111 25404286 PMC4260594

[23] Li F , Bai Y , Guan Z , et al. Dexmedetomidine attenuates sepsis‐associated acute lung injury by regulating macrophage efferocytosis through the ROS/ADAM10/AXL pathway. Int Immunopharmacol. 2024;142(pt A):112832. doi:10.1016/j.intimp.2024.112832 39362816

[24] Yang Y , Wang H , Kouadir M , Song H , Shi F . Recent advances in the mechanisms of NLRP3 inflammasome activation and its inhibitors. Cell Death Dis. 2019;10(2):128. doi:10.1038/s41419-019-1413-8 30755589 PMC6372664

[25] Liu Z , Wang M , Wang X , et al. XBP1 deficiency promotes hepatocyte pyroptosis by impairing mitophagy to activate mtDNA‒cGAS‒STING signaling in macrophages during acute liver injury. Redox Biol. 2022;52:102305. doi:10.1016/j.redox.2022.102305 35367811 PMC8971356

[26] Galluzzi L , Baehrecke EH , Ballabio A , et al. Molecular definitions of autophagy and related processes. EMBO J. 2017;36(13):1811‐1836. doi:10.15252/embj.201796697 28596378 PMC5494474

[27] Zhang J , Zhang M , Huo XK , et al. Macrophage inactivation by small molecule wedelolactone via targeting sEH for the treatment of LPS‐induced acute lung injury. ACS Cent Sci. 2023;9(3):440‐456. doi:10.1021/acscentsci.2c01424 36968547 PMC10037491

[28] Ning L , Wei W , Wenyang J , Rui X , Qing G . Cytosolic DNA‒STING‒NLRP3 axis is involved in murine acute lung injury induced by lipopolysaccharide. Clin Transl Med. 2020;10(7):e228. doi:10.1002/ctm2.228 33252860 PMC7668192

[29] Jiang T , Liu E , Li Z , et al. SIRT1‒Rab7 axis attenuates NLRP3 and STING activation through late endosomal‐dependent mitophagy during sepsis‐induced acute lung injury. Int J Surg. 2024;110(5):2649‐2668. doi:10.1097/js9.0000000000001215 38445453 PMC11093444

[30] Liu N , Pang X , Zhang H , Ji P . The cGAS‒STING pathway in bacterial infection and bacterial immunity. Front Immunol. 2021;12:814709. doi:10.3389/fimmu.2021.814709 35095914 PMC8793285

[31] Zhong W , Rao Z , Rao J , et al. Aging aggravated liver ischemia and reperfusion injury by promoting STING‐mediated NLRP3 activation in macrophages. Aging Cell. 2020;19(8):e13186. doi:10.1111/acel.13186 32666684 PMC7431827

[32] Li N , Zhou H , Wu H , et al. STING‒IRF3 contributes to lipopolysaccharide‐induced cardiac dysfunction, inflammation, apoptosis and pyroptosis by activating NLRP3. Redox Biol. 2019;24:101215. doi:10.1016/j.redox.2019.101215 31121492 PMC6529775

[33] Simovic I , Hilmi I , Ng RT , et al. ATG16L1 rs2241880/T300A increases susceptibility to perianal Crohn's disease: an updated meta‐analysis on inflammatory bowel disease risk and clinical outcomes. United Eur Gastroenterol J. 2024;12(1):103‐121. doi:10.1002/ueg2.12477 PMC10859713 37837511

[34] Li Y , Zhou D , Ren Y , et al. Mir223 restrains autophagy and promotes CNS inflammation by targeting ATG16L1. Autophagy. 2019;15(3):478‐492. doi:10.1080/15548627.2018.1522467 30208760 PMC6351131

[35] Rubio I , Osuchowski MF , Shankar‐Hari M , et al. Current gaps in sepsis immunology: new opportunities for translational research. Lancet Infect Dis. 2019;19(12):e422‐e436. doi:10.1016/s1473-3099(19)30567-5 31630991

[36] Ye C , Li H , Bao M , Zhuo R , Jiang G , Wang W . Alveolar macrophage‐derived exosomes modulate severity and outcome of acute lung injury. Aging. 2020;12(7):6120‐6128. doi:10.18632/aging.103010 32259794 PMC7185135

[37] Cakarova L , Marsh LM , Wilhelm J , et al. Macrophage tumor necrosis factor‐alpha induces epithelial expression of granulocyte‐macrophage colony‐stimulating factor: impact on alveolar epithelial repair. Am J Respir Crit Care Med. 2009;180(6):521‐532. doi:10.1164/rccm.200812-1837OC 19590023

[38] Fu J , Wu H . Structural mechanisms of NLRP3 inflammasome assembly and activation. Annu Rev Immunol. 2023;41:301‐316. doi:10.1146/annurev-immunol-081022-021207 36750315 PMC10159982

[39] Sefik E , Qu R , Junqueira C , et al. Inflammasome activation in infected macrophages drives COVID‐19 pathology. Nature. 2022;606(7914):585‐593. doi:10.1038/s41586-022-04802-1 35483404 PMC9288243

[40] Ying Y , Mao Y , Yao M . NLRP3 inflammasome activation by microRNA‐495 promoter methylation may contribute to the progression of acute lung injury. Mol Ther Nucleic Acids. 2019;18:801‐814. doi:10.1016/j.omtn.2019.08.028 31734560 PMC6861628

[41] Sorbara MT , Ellison LK , Ramjeet M , et al. The protein ATG16L1 suppresses inflammatory cytokines induced by the intracellular sensors Nod1 and Nod2 in an autophagy‐independent manner. Immunity. 2013;39(5):858‐873. doi:10.1016/j.immuni.2013.10.013 24238340

[42] Pizzino G , Irrera N , Cucinotta M , et al. Oxidative stress: harms and benefits for human health. Oxid Med Cell Longev. 2017;2017:8416763. doi:10.1155/2017/8416763 28819546 PMC5551541

[43] Kasai S , Shimizu S , Tatara Y , Mimura J , Itoh K . Regulation of Nrf2 by mitochondrial reactive oxygen species in physiology and pathology. Biomolecules. 2020;10(2):320. doi:10.3390/biom10020320 32079324 PMC7072240

[44] Liu B , Chen Y , St Clair DK . ROS and p53: a versatile partnership. Free Radic Biol Med. 2008;44(8):1529‐1535. doi:10.1016/j.freeradbiomed.2008.01.011 18275858 PMC2359898

[45] Cai J , Pires KM , Ferhat M , et al. Autophagy ablation in adipocytes induces insulin resistance and reveals roles for lipid peroxide and Nrf2 signaling in adipose‐liver crosstalk. Cell Rep. 2018;25(7):1708‐1717.e5. doi:10.1016/j.celrep.2018.10.040 30428342 PMC6802939

[46] Ni HM , Bockus A , Boggess N , Jaeschke H , Ding WX . Activation of autophagy protects against acetaminophen‐induced hepatotoxicity. Hepatology. 2012;55(1):222‐232. doi:10.1002/hep.24690 21932416 PMC3245329

[47] Herb M , Schramm M . Functions of ROS in macrophages and antimicrobial immunity. Antioxidants. 2021;10(2):313. doi:10.3390/antiox10020313 33669824 PMC7923022

[48] Heid ME , Keyel PA , Kamga C , Shiva S , Watkins SC , Salter RD . Mitochondrial reactive oxygen species induces NLRP3‐dependent lysosomal damage and inflammasome activation. J Immunol. 2013;191(10):5230‐5238. doi:10.4049/jimmunol.1301490 24089192 PMC3833073

[49] Ornatowski W , Lu Q , Yegambaram M , et al. Complex interplay between autophagy and oxidative stress in the development of pulmonary disease. Redox Biol. 2020;36:101679. doi:10.1016/j.redox.2020.101679 32818797 PMC7451718

[50] Fritz T , Niederreiter L , Adolph T , Blumberg RS , Kaser A . Crohn's disease: NOD2, autophagy and ER stress converge. Gut. 2011;60(11):1580‐1588. doi:10.1136/gut.2009.206466 21252204 PMC3897479

[51] Conway KL , Kuballa P , Song JH , et al. Atg16l1 is required for autophagy in intestinal epithelial cells and protection of mice from Salmonella infection. Gastroenterology. 2013;145(6):1347‐1357. doi:10.1053/j.gastro.2013.08.035 23973919 PMC3840157

[52] Tschurtschenthaler M , Adolph TE , Ashcroft JW , et al. Defective ATG16L1‐mediated removal of IRE1α drives Crohn's disease‐like ileitis. J Exp Med. 2017;214(2):401‐422. doi:10.1084/jem.20160791 28082357 PMC5294857

[53] Wang L , Cai J , Zhao X , et al. Palmitoylation prevents sustained inflammation by limiting NLRP3 inflammasome activation through chaperone‐mediated autophagy. Mol Cell. 2023;83(2):281‐297.e10. doi:10.1016/j.molcel.2022.12.002 36586411

[54] Ablasser A , Chen ZJ . cGAS in action: expanding roles in immunity and inflammation. Science. 2019;363(6431):eaat8657. doi:10.1126/science.aat8657 30846571

[55] Decout A , Katz JD , Venkatraman S , Ablasser A . The cGAS‒STING pathway as a therapeutic target in inflammatory diseases. Nat Rev Immunol. 2021;21(9):548‐569. doi:10.1038/s41577-021-00524-z 33833439 PMC8029610

[56] Liu S , Yang B , Hou Y , et al. The mechanism of STING autoinhibition and activation. Mol Cell. 2023;83(9):1502‐1518.e10. doi:10.1016/j.molcel.2023.03.029 37086726

[57] Jiang M , Chen P , Wang L , et al. cGAS‒STING, an important pathway in cancer immunotherapy. J Hematol Oncol. 2020;13(1):81. doi:10.1186/s13045-020-00916-z 32571374 PMC7310007

[58] Zhao J , Zhen N , Zhou Q , et al. NETs promote inflammatory injury by activating cGAS‒STING pathway in acute lung injury. Int J Mol Sci. 2023;24(6):5125. doi:10.3390/ijms24065125 36982193 PMC10049640

[59] Gonugunta VK , Sakai T , Pokatayev V , et al. Trafficking‐mediated STING degradation requires sorting to acidified endolysosomes and can be targeted to enhance anti‐tumor response. Cell Rep. 2017;21(11):3234‐3242. doi:10.1016/j.celrep.2017.11.061 29241549 PMC5905341

[60] Gentili M , Liu B , Papanastasiou M , et al. ESCRT‐dependent STING degradation inhibits steady‐state and cGAMP‐induced signalling. Nat Commun. 2023;14(1):611. doi:10.1038/s41467-023-36132-9 36739287 PMC9899276

[61] Gui X , Yang H , Li T , et al. Autophagy induction via STING trafficking is a primordial function of the cGAS pathway. Nature. 2019;567(7747):262‐266. doi:10.1038/s41586-019-1006-9 30842662 PMC9417302

[62] Xu Y , Wang Q , Wang J , et al. The cGAS‒STING pathway activates transcription factor TFEB to stimulate lysosome biogenesis and pathogen clearance. Immunity. 2025;58(2):309‐325.e6. doi:10.1016/j.immuni.2024.11.017 39689715

[63] Fischer TD , Wang C , Padman BS , Lazarou M , Youle RJ . STING induces LC3B lipidation onto single‐membrane vesicles via the V‐ATPase and ATG16L1‐WD40 domain. J Cell Biol. 2020;219(12):e202009128. doi:10.1083/jcb.202009128 33201170 PMC7716379

[64] Wang Q , Bu Q , Liu M , et al. XBP1‐mediated activation of the STING signalling pathway in macrophages contributes to liver fibrosis progression. JHEP Rep. 2022;4(11):100555. doi:10.1016/j.jhepr.2022.100555 36185574 PMC9520276

[65] Wang Q , Bu Q , Xu Z , et al. Macrophage ATG16L1 expression suppresses metabolic dysfunction‐associated steatohepatitis progression by promoting lipophagy. Clin Mol Hepatol. 2024;30(3):515‐538. doi:10.3350/cmh.2024.0107 38726504 PMC11261221

[66] Younes OA , Elsherbiny DM , Hanna DMF , Gad AM , Azab SS . Tocilizumab unfolds colo‐protective and immunomodulatory effect in experimentally induced ulcerative colitis via mitigating autophagy and ER stress signaling. Inflammopharmacology. 2024;32(6):3881‐3898. doi:10.1007/s10787-024-01527-7 39134818 PMC11550239

[67] Cao T , Li AQ , Zhang Y , et al. Norwogonin attenuates LPS‐induced acute lung injury through inhibiting Src/AKT1/NF‐κB signaling pathway. Phytomedicine. 2025;139:156432. doi:10.1016/j.phymed.2025.156432 39922147

[68] Chen P , Wang Y , Tang H , et al. New applications of clioquinol in the treatment of inflammation disease by directly targeting arginine 335 of NLRP3. J Pharm Anal. 2025;15(1):101069. doi:10.1016/j.jpha.2024.101069 39902456 PMC11788862

[69] Zeng QQ , Wang J , Yue RC , et al. Gelsevirine ameliorates sepsis‐associated encephalopathy by inhibiting the STING signalling‐mediated pyroptosis pathway in microglia. Phytomedicine. 2024;135:156071. doi:10.1016/j.phymed.2024.156071 39326131

[70] Hui S , Kan W , Qin S , et al. Glycyrrhiza uralensis polysaccharides ameliorates cecal ligation and puncture‐induced sepsis by inhibiting the cGAS‒STING signaling pathway. Front Pharmacol. 2024;15:1374179. doi:10.3389/fphar.2024.1374179 38904004 PMC11188434

