# Supplementary figures and images for: ATG16L1 restrains macrophage NLRP3 activation and alveolar epithelial cell injury during septic lung injury
Source: Clin Transl Med. 2025 Apr 11;15(4):e70289. doi: 10.1002/ctm2.70289 (PMC11986372; doi:10.1002/ctm2.70289)

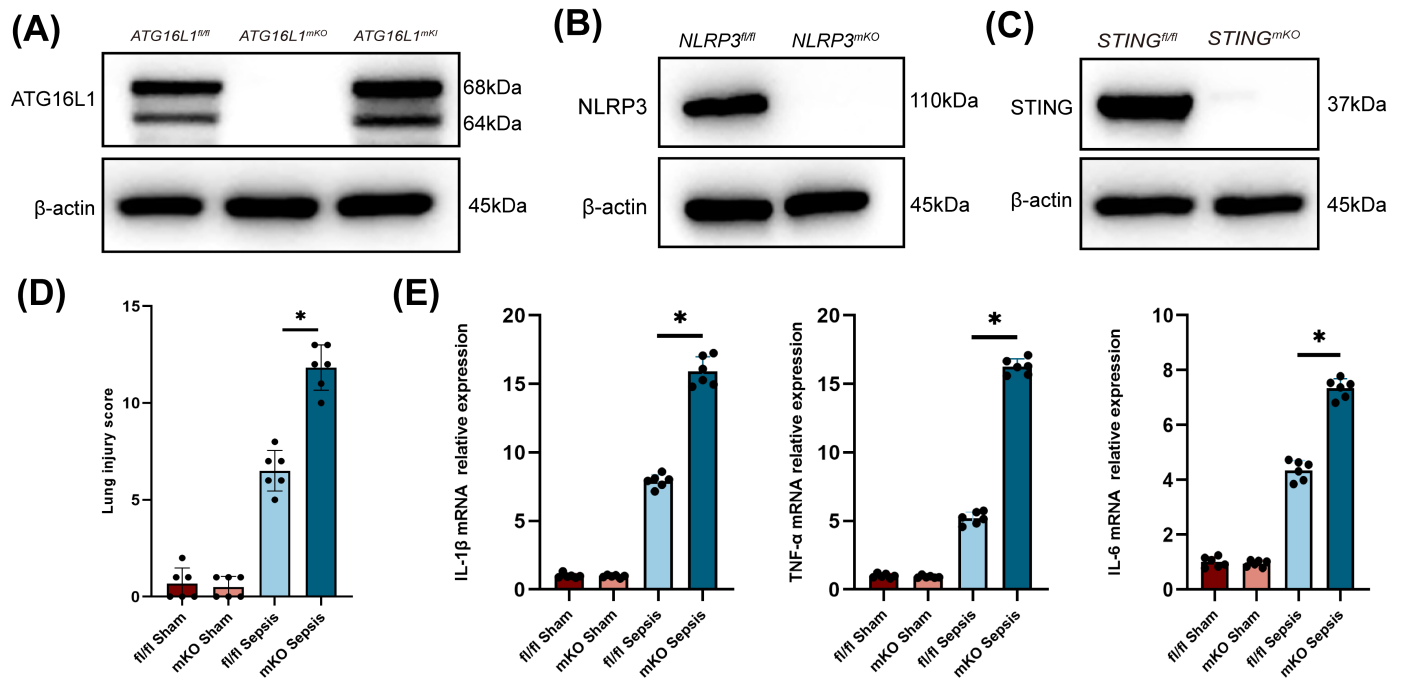

Supplement: Supplementary file 1 — FIGURE S1 Myeloid‐specific ATG16L1 deficiency exacerbates sepsis‐induced lung injury. (A‒C) Western blot analysis of ATG16L1 (A), NLRP3 (B), STING (C) and β‐actin proteins in bone marrow‐derived macrophages (BMDMs). (D) Semiquantitative analysis of lung tissues was performed based on lung injury scores (n = 6/group). (E) Relative expression of IL‐1β, TNF‐α and IL‐6 mRNA in lung tissues detected by RT‐qPCR (n = 6/group). Data are presented as the mean ± SEM. * p < .05. [file CTM2-15-e70289-s003.pdf]

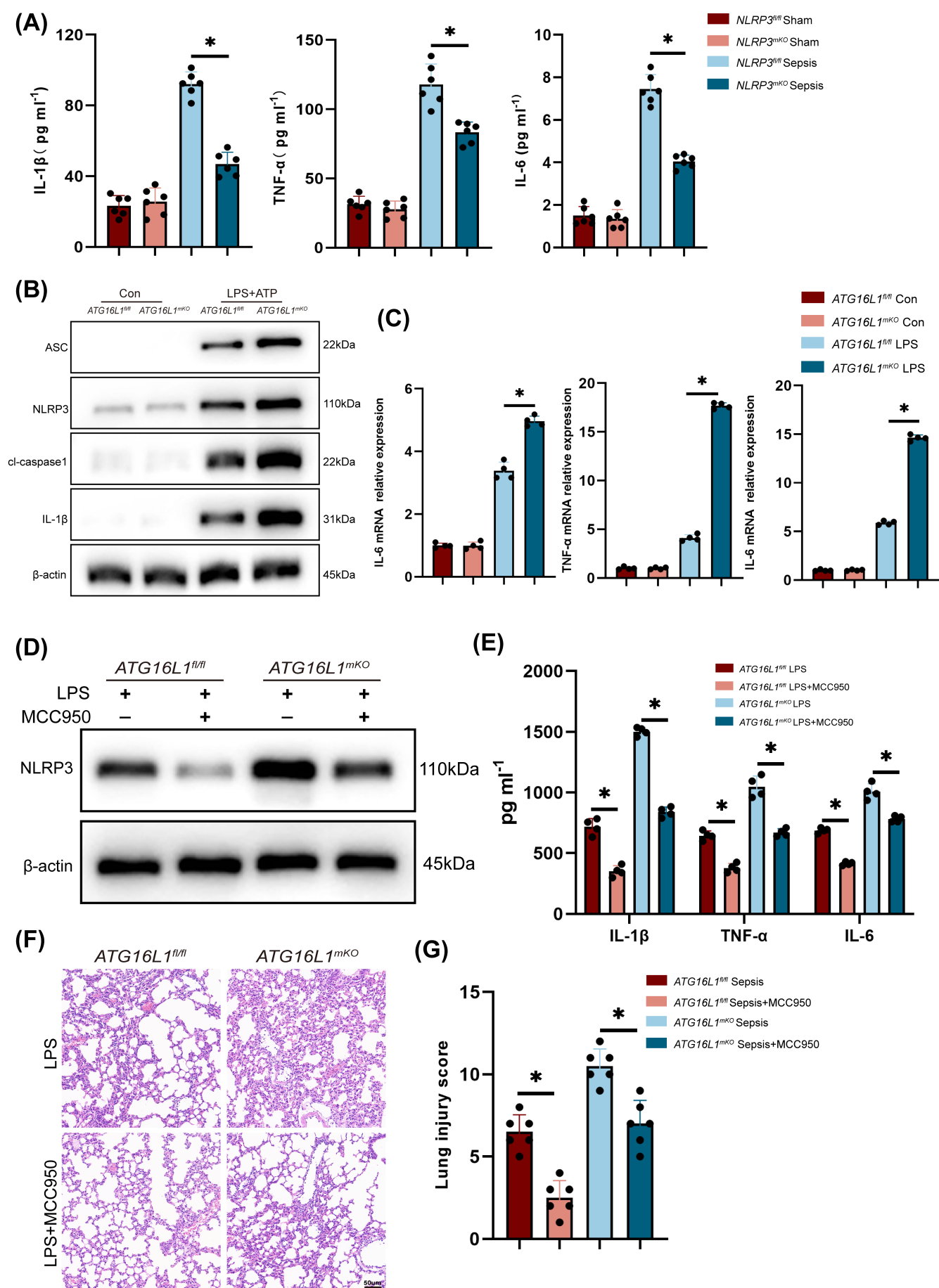

Supplement: Supplementary file 2 — FIGURE S2 ATG16L1 deficiency promotes macrophage NLRP3 inflammasome activation to aggravate acute lung injury (ALI). (A) Concentration of inflammatory cytokines IL‐1β, TNF‐α and IL‐6 in NLRP3fl/fl and NLRP3mKO murine BALF (n = 6/group). (B) Western blot analysis of ASC, NLRP3, cl‐caspase1, IL‐1β and β‐actin in lipopolysaccharide (LPS)‐treated ATG16L1fl/fl and ATG16L1mKO bone marrow‐derived macrophages (BMDMs) (n = 4/group). (C) Relative mRNA expression levels of IL‐1β, TNF‐α and IL‐6 in ATG16L1fl/fl and ATG16L1mKO BMDMs (n = 4/group). (D) Before treating ATG16L1fl/fl and ATG16L1mKO BMDMs with LPS, pretreated with the NLRP3 inhibitor MCC950 (10 µM) for 6 h. Western blot images of NLRP3 and β‐actin in BMDMs (n = 4/group). (E) Levels of inflammatory cytokines IL‐1β, TNF‐α and IL‐6 in the supernatants of BMDMs with MCC950 treatment. (F and G) MCC950 (10 mg/kg) was administered 12 h before LPS‐induced ATG16L1fl/fl and ATG16L1mKO mice sepsis modelling. Haematoxylin and eosin (H&E) staining of lung tissue sections and lung tissues injury scores (n = 6/group; scale bar: 50 µm). Data are presented as the mean ± SEM. * p < .05. [file CTM2-15-e70289-s002.pdf]

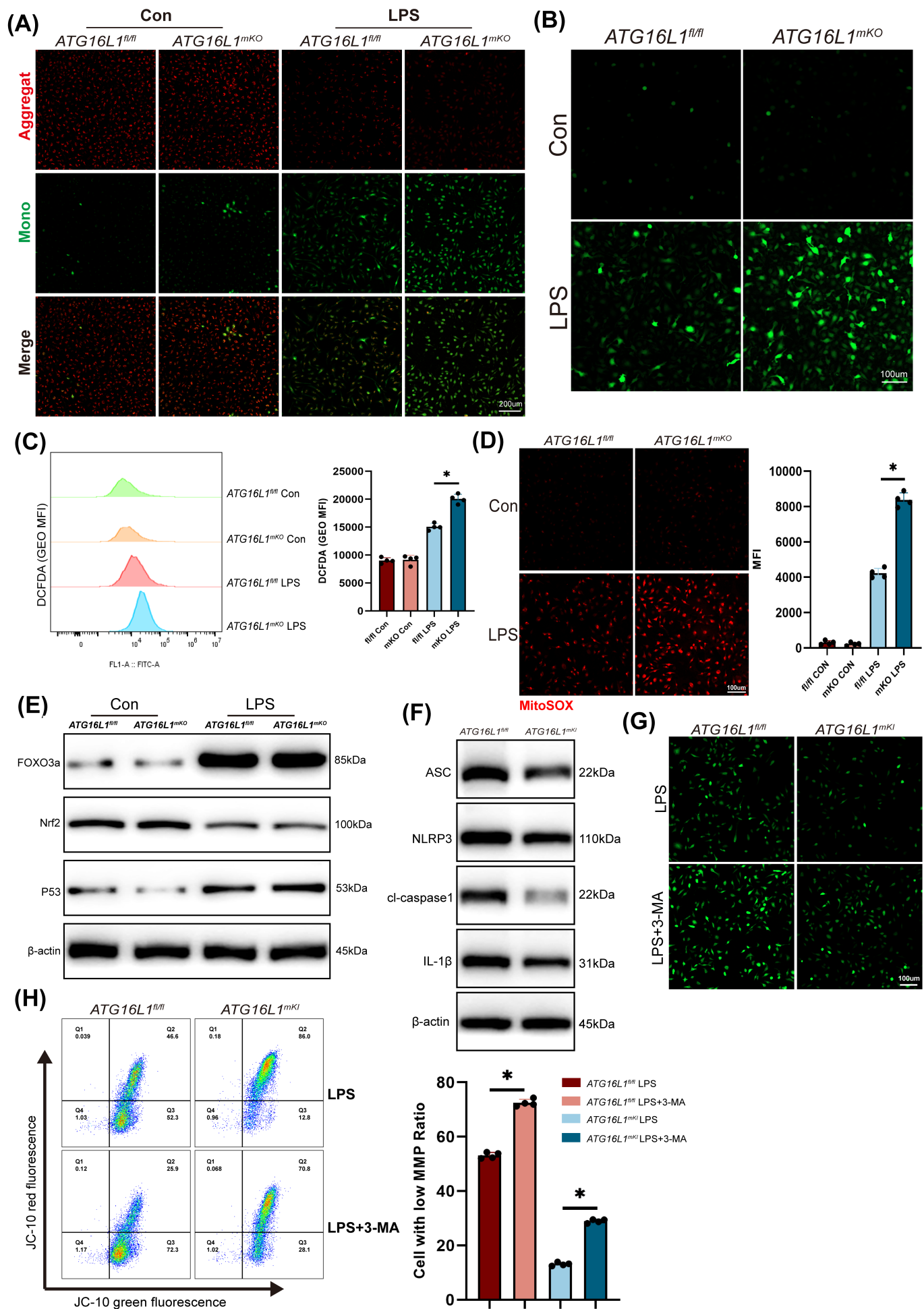

Supplement: Supplementary file 3 — FIGURE S3 Deletion of ATG16L1 increases macrophage reactive oxygen species (ROS) accumulation by inhibiting autophagy. (A) Immunofluorescence detection of mitochondrial membrane potential in ATG16L1fl/fl and ATG16L1mKO bone marrow‐derived macrophages (BMDMs) (n = 4/group; scale bar: 200 µm). (B and C) Levels of intracellular ROS in ATG16L1fl/fl and ATG16L1mKO BMDMs detected by DCFDA fluorescence probe. Representative immunofluorescence images of ROS (B) (scale bar: 100 µm). Representative flow cytometry plot of ROS (C). (D) Mitochondrial reactive oxygen species levels detected by MitoSOX Red fluorescence probe (n = 4/group; scale bar: 100 µm). (E) Western blot analysis of FOXOa3, Nrf2, P53 and β‐actin in ATG16L1fl/fl and ATG16L1mKO BMDMs (n = 4/group). (F) Western blot analysis of ASC, NLRP3, cl‐caspase1, IL‐1β and β‐actin in lipopolysaccharide (LPS)‐treated ATG16L1fl/fl and ATG16L1mKI BMDMs (n = 4/group). (G) Representative fluorescence images of ROS in ATG16L1fl/fl and ATG16L1mKI BMDMs treated with 3‐MA (n = 4/group). (H) Mitochondrial membrane potential detected by flow cytometry in ATG16L1fl/fl and ATG16L1mKI BMDMs treated with 3‐MA (n = 4/group). Data are presented as the mean ± SEM. * p < .05. [file CTM2-15-e70289-s007.pdf]

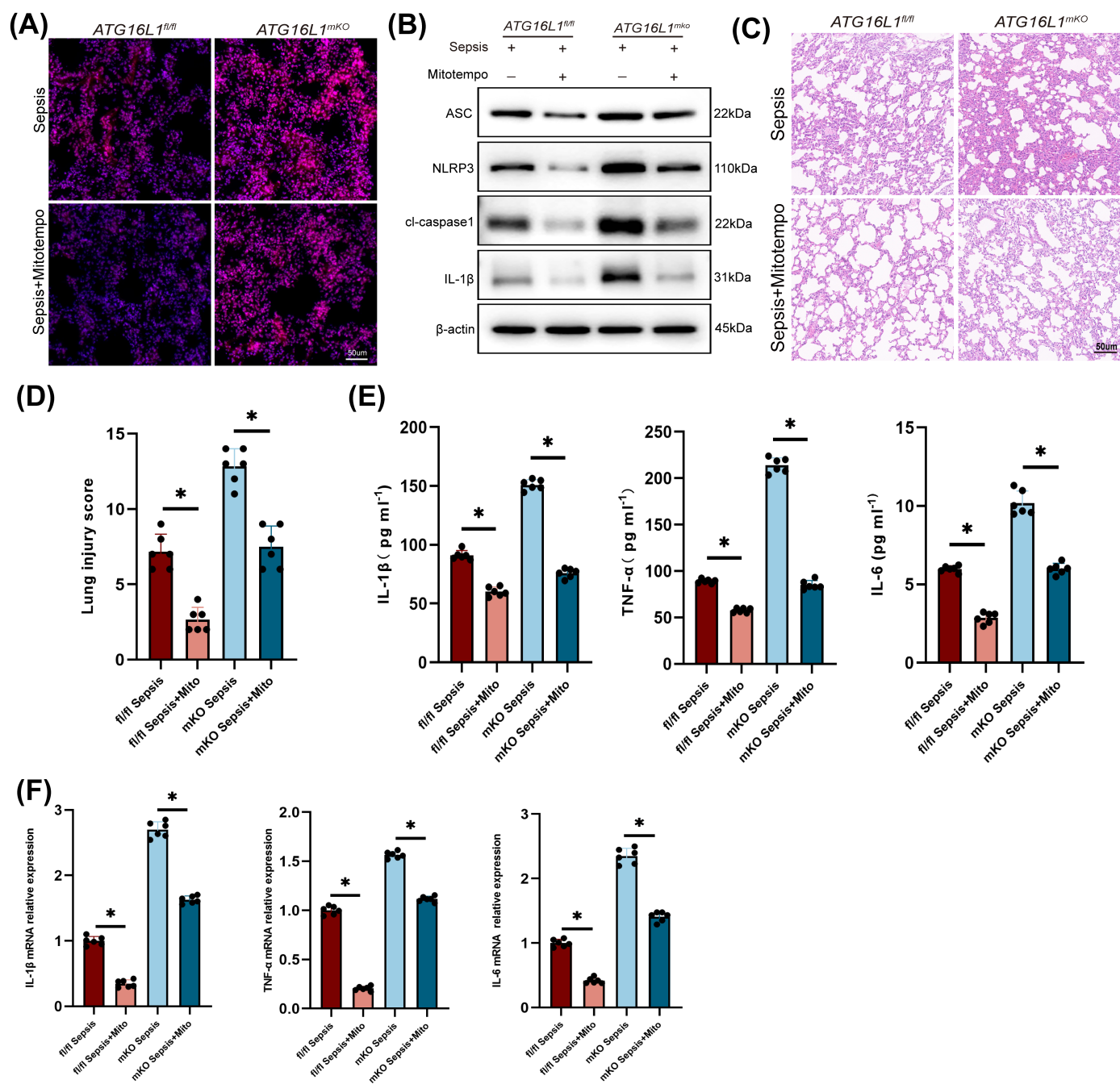

Supplement: Supplementary file 4 — FIGURE S4 Clearance of reactive oxygen species (ROS) inhibits the activation of NLRP3 and improves lung tissues inflammation. (A) Representative images of ROS fluorescence assay in lung tissue using the DHE fluorescent probe (red) in ATG16L1fl/fl and ATG16L1mKO septic mice, with or without Mitotempo pretreatment. Blue indicates nuclei stained with DAPI (n = 6/group; scale bar: 50 µm). (B) Western blot analysis of ASC, NLRP3, cl‐caspase1, IL‐1β and β‐actin in ATG16L1 fl/fl and ATG16L1 mKO septic mice lung tissues treated with Mitotempo (n = 6/group). (C and D) Haematoxylin and eosin (H&E) staining of lung tissue sections and lung tissues injury scores (n = 6/group; scale bar: 50 µm). (E) Levels of inflammatory cytokines IL‐1β, TNF‐α and IL‐6 in ATG16L1 fl/fl and ATG16L1 mKO septic mice BALF (n = 6/group). (F) Relative expression of IL‐1β, TNF‐α and IL‐6 mRNA in lung tissues treated with Mitotempo (n = 6/group). Data are presented as the mean ± SEM. * p < .05. [file CTM2-15-e70289-s008.pdf]

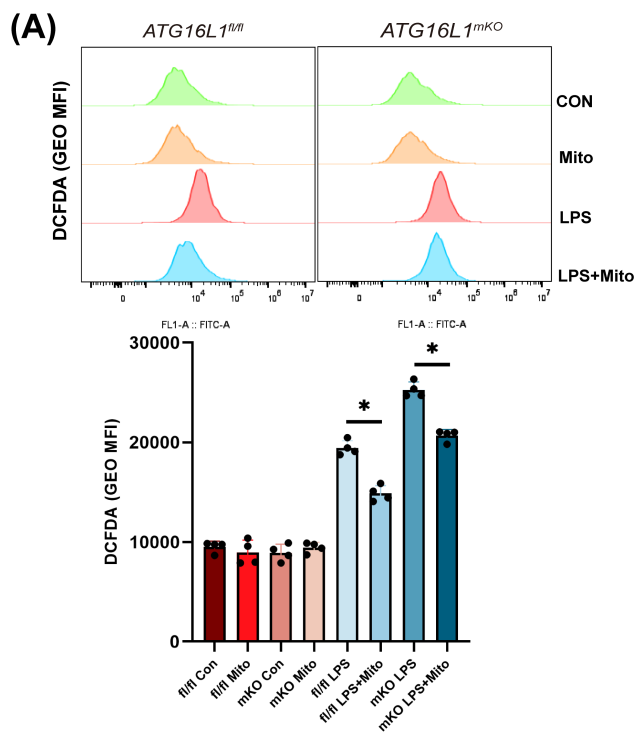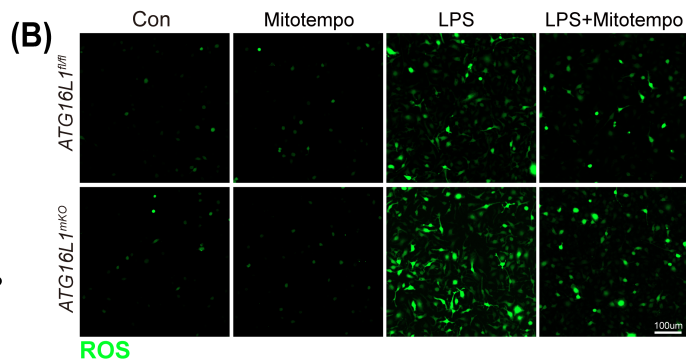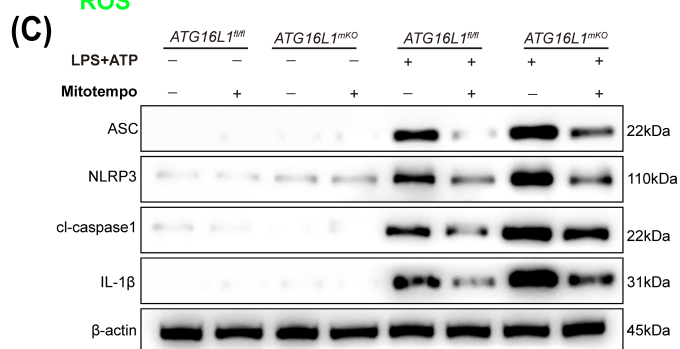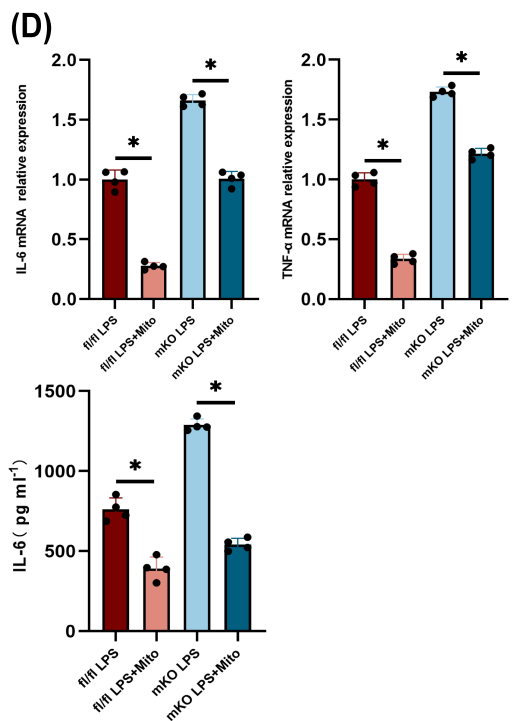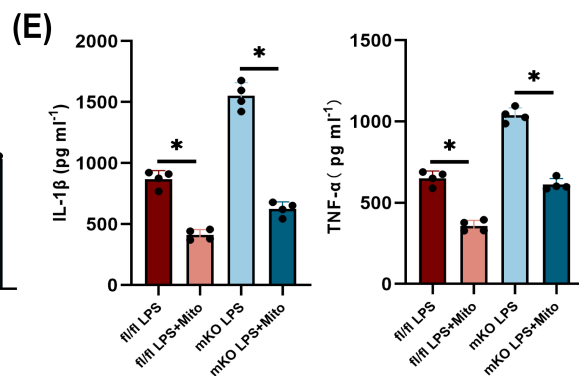

Supplement: Supplementary file 5 — FIGURE S5 Clearance of reactive oxygen species (ROS) suppresses bone marrow‐derived macrophages (BMDMs) inflammatory activation. (A) Intracellular ROS levels detected by flow cytometry in ATG16L1fl/fl and ATG16L1mKO BMDMs Mitotempo pretreatment (n = 4/group). (B) Representative immunofluorescence images of ROS detected by DCFDA fluorescence probe in ATG16L1fl/fl and ATG16L1mKO BMDMs with Mitotempo treatment (n = 4/group; scale bar: 100 µm). (C) Western blot analysis of ASC, NLRP3, cl‐caspase1, IL‐1β and β‐actin in ATG16L1 fl/fl and ATG16L1 mKO BMDMs treated with Mitotempo (n = 4/group). (D) Concentration of inflammatory cytokines IL‐1β, TNF‐α and IL‐6 in the supernatants of BMDMs with Mitotempo treatment (n = 4/group). (E) Relative expression of IL‐1β, TNF‐α and IL‐6 mRNA in BMDMs treated with Mitotempo (n = 4/group). Data are presented as the mean ± SEM. * p < .05. [file CTM2-15-e70289-s004.pdf]

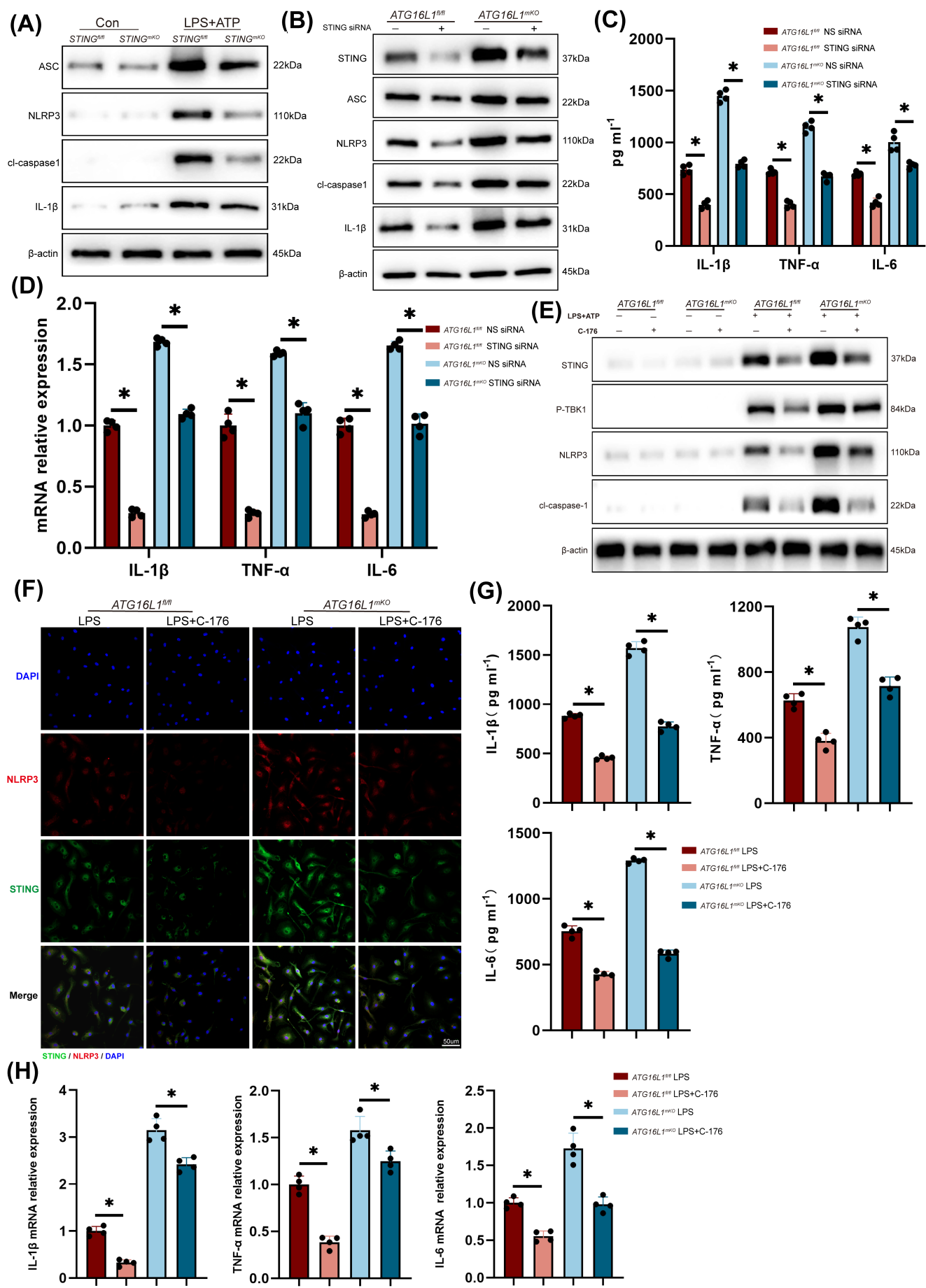

Supplement: Supplementary file 6 — FIGURE S6 Activation of the STING signalling pathway promotes the activation of the NLRP3 inflammasome. (A) Bone marrow‐derived macrophages (BMDMs) were isolated from STINGfl/fl and STINGmKO mice. Representative Western blot analysis of ASC, NLRP3, cl‐caspase1, IL‐1β and β‐actin in BMDMs after lipopolysaccharide (LPS) stimulation. (B) BMDMs were transfected with STING siRNA or non‐specific siRNA (control) and then stimulated with 100 ng/mL of LPS. Protein levels of STING, ASC, NLRP3, cl‐caspase1, IL‐1β and β‐actin were measured using Western blotting (n = 4/group). (C and D) Levels of inflammatory cytokines IL‐1β, TNF‐α and IL‐6 in the supernatants of BMDMs transfected with STING siRNA (n = 4/group). (E) Western blot analysis of STING, P‐TBK1, NLRP3, cl‐caspase1 and β‐actin in ATG16L1fl/fl and ATG16L1mKO BMDMs pretreated with the STING inhibitor C‐176 (n = 4/group). (F) Immunofluorescence staining showed the activation and co‐localisation of NLRP3 (red) and STING (green) after pretreatment with STING inhibitor C‐176. Blue indicates nuclei stained with DAPI (n = 4/group, scale bar: 50 µm). (G and H) Levels of inflammatory cytokines IL‐1β, TNF‐α and IL‐6 in the supernatants of BMDMs pretreated with the STING inhibitor C‐176 (n = 4/group). Data are presented as the mean ± SEM. * p < .05. [file CTM2-15-e70289-s001.pdf]

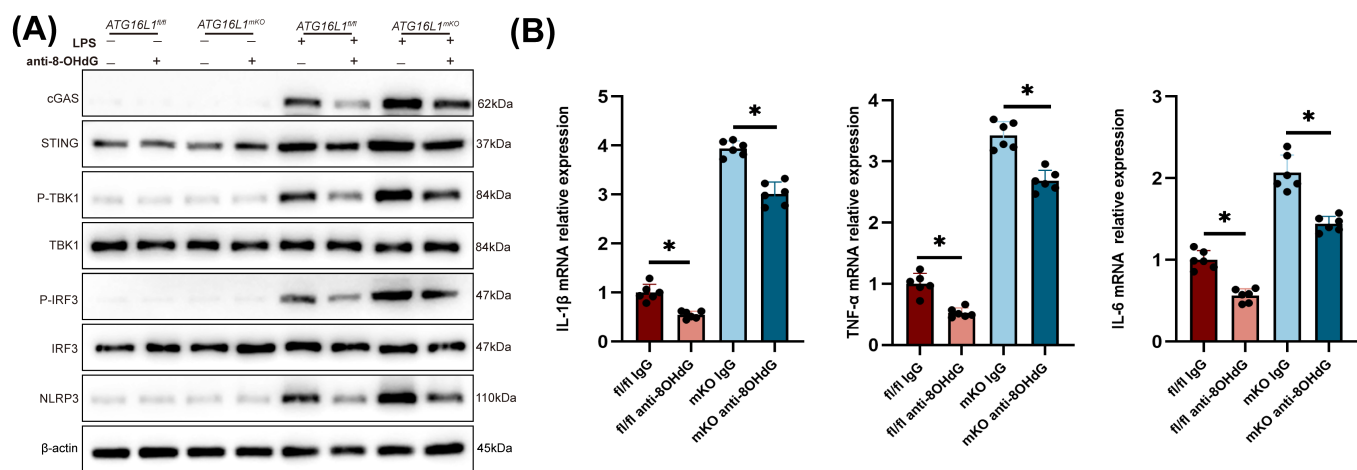

Supplement: Supplementary file 7 — FIGURE S7 Clearance of double‐stranded DNA (dsDNA) suppresses macrophage STING‒NLRP3 activation to protect lungs against septic injury. (A) Western blot analysis of cGAS, STING, P‐TBK1, TBK1, P‐IRF3, IRF3, NLRP3 and β‐actin in bone marrow‐derived macrophages (BMDMs) after treatment with anti‐8‐OHG antibody (n = 4/group). (B) Relative mRNA expression levels of IL‐1β, TNF‐α and IL‐6 in lung tissues after treatment with anti‐8‐OHG antibody (n = 6/group). Data are presented as the mean ± SEM. * p < .05. [file CTM2-15-e70289-s006.pdf]
