# Supplementary material for: ATG16L1 restrains macrophage NLRP3 activation and alveolar epithelial cell injury during septic lung injury
Source: Clin Transl Med. 2025 Apr 11;15(4):e70289. doi: 10.1002/ctm2.70289 (PMC11986372; doi:10.1002/ctm2.70289)
Supplement: Supplementary file 8 — Supporting Information [file CTM2-15-e70289-s005.docx]

**Table S1. Immunofluorescent Antibodies**

| **Name** | **Cat no.** | **Supplier** |
| --- | --- | --- |
| anti-ZO-1 | A0659 | Abclonal |
| anti-Occludin | 66378-1-Ig | Proteintech |
| anti-Claudin3 | ab214487 | Abcam |
| anti-LC3B  anti-STING  anti-NLRP3  anti-F4/80  anti-8-OHdG  Anti-mouse IgG  Anti-mouse IgG  Anti-rabbit IgG  Anti-rabbit IgG | ab192890  ab288157  MA5-34969  ab6640  sc-66036  4408  8890  4412  8889 | Abcam  Abcam  Invitrogen  Abcam  Santa Cruz  Cell Signaling Technology  Cell Signaling Technology  Cell Signaling Technology  Cell Signaling Technology |

**Table S2. Western blotting antibodies**

| **Name** | **Cat no.** | **Supplier** |
| --- | --- | --- |
| Anti-ATG16L1  anti-ZO-1 | 8089  A0659 | Cell Signaling Technology  Abclonal |
| anti-occludin | 66378-1-Ig | Proteintech |
| anti-claudin3 | ab214487 | Abcam |
| anti-LC3B  anti-P62  anti-STING  anti-NLRP3  anti-ASC  anti- cl-caspase1  anti- IL-1B  anti-cGAS  anti-P-TBK1  anti-TBK1  anti-P-IRF3  anti-IRF3  anti- FoxO3a  anti-P53  anti- NRF2  anti-β-actin  Anti-mouse IgG  Anti-rabbit IgG | ab192890  ab109012  ab288157  15101  67824  4199  12242  79978  5483  38066  29047  4302  12829  2527  12721  4967  7076  7074 | Abcam  Abcam  Abcam  Cell Signaling Technology  Cell Signaling Technology  Cell Signaling Technology  Cell Signaling Technology  Cell Signaling Technology  Cell Signaling Technology  Cell Signaling Technology  Cell Signaling Technology  Cell Signaling Technology  Cell Signaling Technology  Cell Signaling Technology  Cell Signaling Technology  Cell Signaling Technology  Cell Signaling Technology  Cell Signaling Technology |

**Table S3. Primers used in this study (M, denotes** **mice)**

| **Gene Symbol** | **Forward Primer** | **Reverse Primer** |
| --- | --- | --- |
| M-Il1b | GCAACTGTTCCTGAACTCAACT | ATCTTTTGGGGTCCGTCAACT |
| M-Tnfa  M-Il6  M-β-Actin | CCCTCACACTCAGATCATCTTCT  TAGTCCTTCCTACCCCAATTTCC  GTGACGTTGACATCCGTAAAGA | GCTACGACGTGGGCTACAG  TTGGTCCTTAGCCACTCCTTC  GCCGGACTCATCGTACTCC |
